# Evidence of Some Natural Products with Antigenotoxic Effects. Part 2: Plants, Vegetables, and Natural Resin

**DOI:** 10.3390/nu10121954

**Published:** 2018-12-10

**Authors:** David López-Romero, Jeannett A. Izquierdo-Vega, José Antonio Morales-González, Eduardo Madrigal-Bujaidar, Germán Chamorro-Cevallos, Manuel Sánchez-Gutiérrez, Gabriel Betanzos-Cabrera, Isela Alvarez-Gonzalez, Ángel Morales-González, Eduardo Madrigal-Santillán

**Affiliations:** 1 Instituto de Ciencias de la Salud, Universidad Autónoma del Estado de Hidalgo, Ex-Hacienda de la Concepción, Tilcuautla, Pachuca de Soto 42080, Hgo, Mexico; david.daloro@gmail.com (D.L.-R.); jizquierdovega@gmail.com (J.A.I.-V.); spmtz68@yahoo.com.mx (M.S.-G.); gbetanzo@uaeh.edu.mx (G.B.-C.); 2 Escuela Superior de Medicina, Instituto Politécnico Nacional, “Unidad Casco de Santo Tomas”. Plan de San Luis y Díaz Mirón s/n, Ciudad de México 11340, Mexico; jmorales101@yahoo.com.mx; 3 Escuela Nacional de Ciencias Biológicas, Instituto Politécnico Nacional, “Unidad Profesional A. López Mateos”. Av. Wilfrido Massieu. Col., Lindavista, Ciudad de México 07738, Mexico; eduardo.madrigal@lycos.com (E.M.-B.); gchamcev@yahoo.com.mx (G.C.-C.); isela.alvarez@gmail.com (I.A.-G.); 4 Escuela Superior de Cómputo, Instituto Politécnico Nacional, “Unidad Profesional A. López Mateos”. Av. Juan de Dios Bátiz. Col., Lindavista, Ciudad de México 07738, Mexico; anmorales@ipn.mx

**Keywords:** antigenotoxic, plants, vegetables, propolis, cancer, micronucleus, comet assay

## Abstract

Cancer is one of the leading causes of death worldwide. The agents capable of causing damage to genetic material are known as genotoxins and, according to their mode of action, are classified into mutagens, carcinogens, or teratogens. Genotoxins are also involved in the pathogenesis of several chronic degenerative diseases, including hepatic, neurodegenerative, and cardiovascular disorders; diabetes; arthritis; cancer; chronic inflammation; and ageing. In recent decades, researchers have found novel bioactive phytocompounds able to counteract the effects of physical and chemical mutagens. Several studies have shown the antigenotoxic potential of different fruits and plants (Part 1). In this review (Part 2), we present a research overview conducted on some plants and vegetables (spirulina, broccoli, chamomile, cocoa, ginger, laurel, marigold, roselle, and rosemary), which are frequently consumed by humans. In addition, an analysis of some phytochemicals extracted from those vegetables and the analysis of a resin (propolis),whose antigenotoxic power has been demonstrated in various tests, including the Ames assay, sister chromatid exchange, chromosomal aberrations, micronucleus, and comet assay, was also performed.

## 1. Introduction

Genotoxicity is the ability of different agents to produce damage to genetic material. However, the damage induced in the genetic material includes not only DNA, but also thecellular components related to the functionality and behavior of chromosomes within the cell. An example of this are the proteins involved in the repair, condensation, and decondensation of DNA in the chromosomes, or other structures, such as the mitotic spindle, responsible for distribution of the chromosomes during cell division [1,2,3]. The agents capable of causing genetic toxicity are described as genotoxic or called genotoxins; and, according to their origin, they are classified into three categories: Physical, chemical, and biological. The first category includes the ionizing and electromagnetic radiation, temperature, and ultraviolet light. The second group consists of a wide range of compounds with multiple effects, highlighting the heavy metals, pesticides, aromatic hydrocarbons, alkylating agents, acridine, acrylamide, aliphatic epoxides, organic solvents, asbestos particles, food additives, and xenobiotics resulting from certain “lifestyles”, such as smoking or drinking (alcoholism). The last category considers some parasites, bacteria, plants, viruses, and fungi (specifically those that synthesize secondary metabolites, such as mycotoxins) [3,4,5].

**Figure 1:**
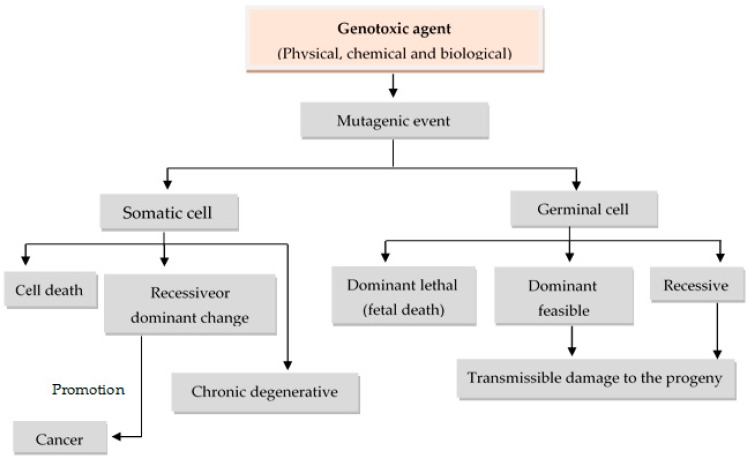
Effects produced by a genotoxic agent [6].

At the same time, genotoxic agents may also be classified according to their effects or mode of action into mutagens, carcinogens, or teratogens, resulting in three types of processes: Mutagenesis, carcinogenesis, and teratogenesis. Mutagenesis considers, basically, two types of genetic alterations. Alterations (mutations) that may occur at the level of a minimum unit of information (gene) or higher-level units, such as structural groups (chromosomes), to what is called micromutation or macromutation, respectively [2,3]. In the case of macromutations, the clastogenic agents are defined as those capable of inducing chromosome breaks, and aneunogen agents are those who produce the loss of whole chromosomes or chromosome sets. Mutations may occur on somatic and/or germ cells, with latter case inheritable if they are transmitted to the progeny. There is increasing evidence that mutation in somatic cells are not only involved in carcinogenesis, but can also cause genetic disorders, such as arteriosclerosis, heart diseases, and several other chronic degenerative diseases (Figure 1) [1,4,6].

Carcinogenesis is a process that involves changes, such as irreversible cell transformation, through a series of stages (initiation, promotion, and progression). It has been observed that 90%–95% of carcinoma cases are associated with chemical agents, 5%–10% with physical agents, and between 2%–5% with biological agents. Moreover, teratogenesis involves induced damage in the organism’s development; that is to say, at any time during the gestation period [2,3,4]. It is important to remember that the ability to induce damage of these genotoxic agents is influenced by the dose, time, or route of exposure, together with the genetic constitution of the individual, which can define susceptibility.

Since the genotoxic agents are involved in the initiation and promotion of several human diseases, the significance of novel bioactive phytocompounds in counteracting these mutagenic and carcinogenic effects is now gaining credence. Such chemicals that reduce the mutagenicity of physical and chemical mutagens are referred to as antimutagens. However, taking into account that all mutagens are genotoxic, but not all genotoxic substances are mutagenic, the compounds that reduce the DNA damage caused by genotoxic agents are also called antigenotoxic agents [1,7,8].

**Figure 2:**
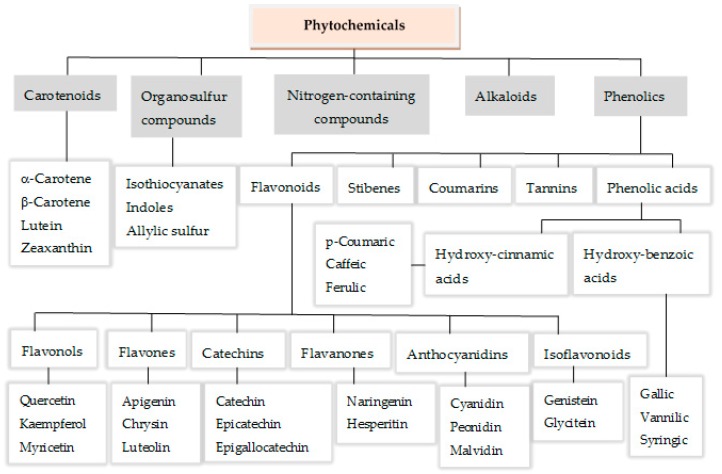
Classification of dietary phytochemicals [9].

Numerous studies have been carried out in last decades in order to identify compounds that might protect humans against DNA damage and its consequences. There are continual efforts all over the world to explore the rich biodiversity of edible (fruits, vegetables) as well as medicinal plants and other edible non-toxic plants in pursuit of the most effective phyto-antimutagens. These bioactive compounds can be classified according to a chemical, biogenetic, or pharmacological criterion. In general, phytochemicals have been organized into five groups, such as carotenoids, phenolic compounds, alkaloids, nitrogen-containing compounds, and organic sulfur compounds (Figure 2) [7,8,9].

Many of these substances, apart from their antimutagenic and anticarcinogenic properties, have shown other beneficial effects for health, such as immunomodulator, hepatoprotective, antihyperglycemic, antihyperlipidemic, cardioprotective, anti-inflammatory, and antirheumatic actions, owing to their excellent antioxidant and detoxifying properties [1].

In general, the antimutagens have been classified as desmutagens and bio-antimutagens; the first group considers substances that promote the elimination of genotoxic agents from the organism as well as substances that inactivate the mutagens partially or fully by enzymatic or chemical interaction before the mutagen attacks the DNA (these must be considered only as apparent antimutagens). On the other hand, bio-antimutagens (also known as true antimutagens) can suppress the process of mutation after DNA is damaged by mutagens. They act on the repair and replication processes of mutagen-damaged DNA, resulting in a decline in mutation frequency [7,8].

The mechanisms of action of the antigenotoxic agents are complex and can be categorized according to the site of action or by the specific type of action. An obvious approach is to avoid exposure to recognized risk factors. However, complementary strategies are to render the organism more resistant to mutagens/carcinogens and/or to inhibit progression of the chronic disease by administering chemopreventive agents. In a primary prevention setting, addressed to apparently healthy individuals, it is possible to inhibit mutation and cancer initiation by triggering protective mechanisms either in the extracellular and intracellular environment, e.g., modifying transmembrane transport, modulating metabolism, blocking reactive species, inhibiting cell replication, maintaining DNA structure, modulating DNA metabolism and repair, and controlling gene expression. Tumor promotion can be counteracted by favoring antioxidant and anti-inflammatory activity, inhibiting proteases and cell proliferation, inducing cell differentiation, and modulating apoptosis and signal transduction pathways. In a secondary prevention setting, when a premalignant lesion has been detected, it is possible to inhibit tumor progression via the same mechanisms or through affecting the hormonal status and the immune system, and inhibiting tumor angiogenesis. Finally, in tertiary prevention (strategy considered outside of the classical definition of chemoprevention) addressed to cancer patients after therapy, similar mechanisms have been explored, highlighting the possibility to affect cell-adhesion molecules and activation of antimetastasis genes [7,10]. The main chemopreventive mechanisms along with some examples of dietary antimutagens are shown in Table 1.

Genetics toxicology is a multidisciplinary science that studies the interaction of physical, chemical, and biological agents with the genetic material, the response mechanisms to the damage, and their impact on the organisms. Due to its wide application in environmental and human monitoring, it has also been used to evaluate the antigenotoxic effects of the plants, vegetables, fruits, and substances of recent formulation. There are different assays, in vitro and in vivo (Table 2), to determine the genoprotector capacity of the compounds; therefore, it would be complicated to describe in detail each one. Each test has its advantages and disadvantages, but, overall, the use of sensitive assessment methods that are rapid, simple, and able to evaluate the genotoxic and antigenotoxic effect on somatic and germ cells as well as proliferating and non-proliferating cells have been considered [2,11,12]. Among the tests that most excelled in the last decades, we can mention the bacterial mutation assay (Ames test), sister chromatid exchange, evaluation of chromosomal aberrations, micronucleus assay, and, more recently, the comet assay or single cell electrophoresis [13,14,15,16,17].

There are countless studies in this field, though all of them would be impossible to mention; however, we can refer to some in order to illustrate the usefulness of the antigenotoxic tools used in the population. This present report aims to gather a good deal of data based on works conducted in some plants, which have demonstrated antigenotoxic capacity, as well as some phytochemicals extracted from vegetables and plants, and that have been evaluated in five of the different models used in genetic toxicology (salmonella mutagenicity test, sister chromatid exchange (SCE), chromosomal aberrations, micronucleus, and comet assay). With these goals in mind, the authors of this paper attempt to provide information and bibliographic support to researchers who can explore the potential of these studies in this area of study.

## 2. Antigenotoxic Plants and Vegetables

### 2.1. Blue Green Algae Spirulina (Arthrospira maxima and Arthrospira Platensis)

**Overview:** Spirulina (classified as *Arthrospira* sp.) is a microscopic blue-green filamentous alga that floats freely on water media; it grows in fresh water, as well as in alkaline salt water. It is a cyanobacterium belonging to the class, Cyanophyceae, and the order, Oscillatoriales. It is an organism capable of storing different bioactive molecules, among which are the following: (1) Proteins (60%–65% dry weight) with essential amino acids; (2) polyunsaturated fatty acids, such as linoleic acid; (3) vitamins (B12 and E); (4) polysaccharides; (5) minerals (Na, K, Ca, Fe, Mn, and Se); and (6) pigments (chlorophyll, C-phycocyanin, allophycocyanin, β-carotene, lutein, and zeaxanthin). Generally, the content of the compounds varies from species-to-species proportions, but the phytochemicals that are always present in their biomass are C-phycocyanin (with a content of 12.6% in dry spirulina) and high percentages of dietary zeaxanthin [18,19]. Spirulina is the sole blue-green alga that is commercially cultivated for food use; there are various species, but those of the greatest commercial importance are *A. maxima* and *A. platensis* (also known as *Spirulina maxima* and *Spirulina platensis*). Until 1989, these species had belonged to the Spirulina genus and their scientific names, respectively, were *S. maxima* and *S. platensis*, both known as “spirulina” [18,19,20].

For centuries, humans have consumed spirulina in many parts of the world, ranking from the Aztec civilization in Latin America to the tribes that inhabit the Lake Chad region of central Africa. In 1996, the World Health Organization declared spirulina the best food for the future because of contemporary scientific studies that have found a high content of proteins and natural vitamins [18,19]. Thus, Spirulina is considered safe for human consumption and has gained popularity both as a food supplement and an important ingredient in many nutraceutical formulations worldwide. Currently, there are numerous scientific evidences of its biological effects against health problems for its antioxidant, anti-inflammatory, hypolipemic, antihypertensive, antidiabetic, antimicrobial, neuroprotective, antianemic, immunostimulant, anticarcinogenic, and hepatoprotective properties [18,19,20,21,22].

**Antigenotoxic evidence for spirulina and its main phytochemical (phycocyanin):** It is curious that, despite spirulina being consumed for centuries, there is only one antigenotoxic study carried out in the eighties, when the radioprotective effect of an extract of *Spirulina platensis* in mouse bone marrow cells was analyzed through the micronucleus test [23]. Probably, the result of observing a significant decrease in the frequency of micronucleated polychromatic erythrocytes induced by gamma radiation was the reason to start different studies on its genoprotective effects in this century.

Specifically, in 2001, the protective effect of *S. fusiformis* on the genotoxicity and oxidative stress induced by cyclophosphamide (CP) and mitomycin C (MMC) in mice, was evaluated. The results showed that a five-day-oral pre-treatment with three doses (250, 500, and 1000 mg/kg) of this alga significantly reduced the chromosomal damage and lipid peroxidation induced by CP and MMC [24]. Subsequently, Madrigal-Bujaidar et al. [25] evaluated the anticlastogenic effect of an extract from *S. maxima* on the micronuclei (MN) induced by maleic hydrazide (MH) in Tradescantia. These researchers used the herbaceous plant as a study model considering that it has six pairs of relatively large chromosomes in their somatic cells that make it a suitable experimental material for micronucleus assessment. Their results showed that when two doses of aqueous extracts of spirulina (100 mg/mL and 500 mg/mL) were applied to Spirulina inflorescences immediately before the application of MH, the genotoxic damage caused by this mutagenic agent was reduced by around 60%. They also observed that no aqueous extracts of spirulina increased the MN level [25].

With respect to the single cell electrophoresis, there are currently only three studies. In one of them, this assay was only used as an evaluation test, while in the others, the genoprotective capacity of these algae was evaluated by means of the comet and micronucleus tests. In the first, the beneficial effect of *S. platensis* on tissue lipid peroxidation and oxidative DNA damage were analyzed with the hypercholesterolemic New Zealand white rabbit model. After adding two concentrations of alga (1% and 5%) to the diet for eight weeks, the supplement showed a significant reduction on the level of lipid peroxidation and DNA damage of lymphocytes in rabbits fed with a cholesterol-enriched diet [26].

In relation to the other studies where both tests (comet and micronucleus assay) were applied, Hassan et al. [27] analyzed the dietary supplementation of spirulina (SPN) on the modulation of DNA damage and the alteration of the gene expression during aflatoxicosis in Sprague-Dawley male rats. They observed that SPN reduced the oxidative stress generated during the disease produced by aflatoxins. Therefore, it was possible to inhibit the damage in the genetic material, confirming a down-regulation of Fas gene expression and a relevant decrease in the percentage of fragmented DNA and micronucleated erythrocytes.

In recent years, Egyptian researchers evaluated the protective capacity of *S. platensis* against a genotoxic agent of physical origin that, nowadays, affects a large number of people in the world; cellular phone radiation. They explored the radioprotective capacity of SPN in adult male Sprague-Dawley rats that were exposed to the effects of GSM 900-MHz cellular phone radiation for 6 h on a daily fashion during 30 days. Mobile phone radiation exposure evoked a marked increase in the frequencies of micronucleated polychromatic erythrocytes in the peripheral blood and bone marrow, in comparison to the control group. Conversely, SPN significantly reduced the level of DNA damage and oxidative stress resulted from electromagnetic phone radiations. These results suggest that a continuous exposure to mobile phone radiation for a long time leads to a significant toxic effect on the bone marrow. Similarly, the oral administration of *S. platensis* can be a useful alternative for radioprotection [28].

The evidence shown in previous studies have suggested that spirulina can be considered a good antigenotoxic. That consideration has motivated other researchers, such as Chamorro-Cevallos and collaborators, to evaluate its genoprotective effect in other experimental models. They analyzed the antimutagenic potential of *S. maxima* against cyclophosphamide [29] and benzo[a]pyrene [30] using the dominant lethal test. In both investigations, mice of both sexes were orally administered spirulina in three doses (200 mg/kg, 400 mg/kg, and 800 mg/kg body weight) for two weeks before the intraperitoneal administration of mutagens for five consecutive days. At the end of the experiment, the results showed that the alga inhibited the genetic damage to germ cells, while it significantly reduced the pre- and postimplant losses in males, and the postimplantation losses in the treated females.

With respect to phycocyanin, the main phytochemical of spirulina, there are only two studies that have shown a possible chemopreventive effect. In the first one, the antioxidant and antiproliferative activity of selenium-containing phycocyanin (Se-PC) purified from *S. platensis* were evaluated in an in vitroexperiment. A possible conclusion of the comet assay was that Se-PC protected erythrocytes from oxidative damage induced by H_2_O_2_ [31]. The purpose of the second study was to determine the inhibitory effect of *S. maxima* (Sm) and its protein extract (PE), mainly constituted by C-phycocyanin, on micronuclei and bone marrow cytotoxicity induced by hydroxyurea (HU) in pregnant mice and their fetuses. At the end of the study, a significant increase in the number of micronucleated polychromatic erythrocytes (MNPE) in all HU-treated animals was observed. In contrast, a low number of MNPE in the groups only treated with Sm and PE and in the lots combined with hydroxyurea plus the alga and its protein extract was also observed [32].

### 2.2. Broccoli (Brassica Oleracea Itálica)

**Overview:** The genus, Brassica, (family Brassicaceae or Cruciferae) includes a high number of vegetables comprising amongst others broccoli, cauliflower, Brussel sprouts, kohlrabi, cabbage, and mustard, which are grown for their edible inflorescences, fleshy stems, roots, or their oils, which are extracted from the seeds. The sprouting broccoli (commonly called broccoli, broccoli florets, or broccoli heads), deriving from *Brassica oleracea* L. var. Italica Plenk, is thought to have originally come from the eastern Mediterranean area and introduced to Europe, especially to Italy, in medieval ages. At present, many cultivars are grown in Europe, Asia, and America [33].

Broccoli is considered one of the most frequently consumed raw vegetables, mainly in fresh salads and soups [34]. According to the United States Department of Agriculture (USDA), broccoli is a rich source of carbohydrates, potassium, vitamin K, vitamin C, vitamin A, vitamin E, potassium, and folate. It is an excellent source of dietary fiber, proteins, calcium, phosphorus, magnesium, and sodium [35]. Due to its pleasant flavor and chemopreventive effects attributed to its glucosinolates and degradation products, broccoli is highly valued by large groups of people. Its important dietary use has encouraged scientists to test a wide range of biological activities, including the gastroprotective, antimicrobial, antioxidant, anticancer, hepatoprotective, cardioprotective, anti-obesity, antidiabetic, anti-inflammatory, and immunomodulatory activities [36,37].

**Antigenotoxic evidence** for **broccoli** and its main phytochemicals (sulforaphane, phenethyl isothiocyanate, allyl isothiocyanate, and indole-3-carbinol): After tomato, carrot, and celery, broccoli is the fourth most frequently consumed vegetable in the world. For this reason, there is great scientific interest in studying its biological effects, especially its DNA protective capacity [38].

Diverse studies have been developed since 1980, all of which have shown the main phytochemicals of broccoli are good candidates to be considered antigenotoxic agents. The results of various studies both in vitro and in vivo suggest that when brocolli is evaluated in different ways (such as broccoli juice, fresh broccoli, broccoli dialysate, deep-frozen commercial broccoli, steamed before being consumed, boiled, or cooked in the microwave, and/or in extracts), there is an antimutagenic activity (strains TA98 and TA100 of *Salmonella typhimurium*); and may reduce the frequency of chromosomal aberrations, sister chromatid exchange (SCE), and micronuclei, as well as single or double strand breaks of DNA produced by benzo(a)pyrene (BaP), aflatoxin B_1_ (AFB_1_), acrylamide, methyl methanesulfonate (MMS), cyclophosphamide (CP), mitomycin C (MMC), N-nitrosodimethylamine (NDMA), N-nitrosopyrrolidine (NPYR), N-nitrosodibutylamine (NDBA) and N-nitrosopiperidine (NPIP), 2-acetylaminofluorene (AAF), N-nitroso-N-methylurea (MNU), hydrogen peroxide (H_2_O_2_), doxorubicin (DXR), gamma-radiation, 3-amino-l-methyl-5H-pyrido (4,3-b) nature (Trp-P-2), N-methyl-N’-nitroso-N-nitrosoguanidine, 2-amino-3-methyl [4,5-f]-quinoline (IQ), 2-amino-3,4-dimethylimidazo [4,5-f] quinoline (MeIQ), 2-amino-3,8-dimethylimidazo [4,5-f] quinoxaline (MeIQx), 2-amino-1-methyl-6-phenylimidazo[4,5-b]pyridine (PhIP), and urethane (Table 3).

With respect to its phytochemicals, more than 120 glucosinolates (GLS) have been characterized so far. All of them share a similar basic structure that consists of a β-d-thioglucose group, a sulphonated oxime group, and a side chain derived from methionine, phenylalanine, tryptophane, or a branched-chain aminoacid. GLSs are not bioactive in the animal that consumes them until they have been enzymatically hydrolysed to an associated isothiocyanate by the endogenous myrosinase enzyme that is released by disruption of the plant cell through harvesting, processing, or mastication. The most characterized and studied GLS compounds are sulphoraphane, phenethyl isothiocyanate, allyl isothiocyanate, and indole-3-carbinol, but many other isothiocyanates that are present in lower quantities may also contribute to the biological chemopreventive properties of broccoli [39,40].

Like broccoli (fresh vegetable or extract), diverse evidence has confirmed the chemoprotective effect of its main phytochemicals (Table 4). In summary, from 1996 until 2013, 10 in vitro studies have been developed (using an Ames test and/or a comet assay), an in vivo experiment (using the micronucleus assay), and a single human study.

In the case of in vitro models, the data have confirmed that glucosinolates (GLS) may inhibit mutagenicity and reduce the DNA single strand break produced by heterocyclic amines, BaP, N-nitrosamines, some pesticides (endosulfan, chlorpyrifos, and thiram), and H_2_O_2_.

Using the micronucleus assay, the mutagenicity of the pesticide propoxur and its inhibition was determined by the administration of indole-3-carbinol (I3C) in Swiss mice. A single intraperitoneal administration propoxur induced the micronucleus formation in bone marrow cells after a 24-h and 48-h exposure. In contrast, the administration of I3C (500 mg/kg body weight) showed a significant reduction of the propoxur-induced MN formation after 48 h of a single application.

In relation to the only clinical study, a controlled intervention study was conducted with 14 participants (10 women and four men) to assess the chemopreventive nature of GLS. Human volunteers were administered daily with GLS for four days. At the end of the period, heparinized blood samples were obtained to evaluate their antigenotoxic potential against hydrogen peroxide using single-cell gel electrophoresis and the micronucleus assay. The previous intake of GLS resulted in a significant reduction of damage on DNA and micronucleus formation induced by H_2_O_2_ [41,42,43,44,45,46,47,48,49,50,51].

### 2.3. Chamomile (Matricaria chamomilla or Chamomilla Recutita)

**Overview:** While there is extensive literature suggesting health benefits associated with drinking teas prepared with *Camellia sinensis* (i.e., black and green teas) [71], evidence-based information regarding the effects of the majority of herbal teas, or tisanes, has been quite limited. One of the most commonly consumed single-ingredient herbal teas is chamomile [72]. Chamomile (*Matricaria chamomilla* or *Chamomilla recutita*) is an Asteraceae plant native to Europe, and is distributed worldwide, except in tropical and polar regions. This plant has been used for its curative properties since the ancient Egyptian and Greek civilizations. Currently, it is frequently used as an antiseptic, antiphlogistic, diuretic, expectorant, febrifuge, sedative, anti-inflammatory, antioxidant, antibacterial, antiviral, antifungal, antispasmodic, spasmolytic, anti-ulcer, antiallergic, and anticarcinogenic [73,74]. Chamomile is a relevant source of natural products. The essential oil extracted from the chamomile flower ranges from 0.42% to 2%; and consists of compounds, such as sesquiterpene derivatives (75% to 90%), (*E*)-β-farnesene (4.9% to 8.1%), terpene alcohol (farnesol), chamazulene (2.3% to 10.9%), α-bisabolol (4.8% to 11.3%), α-bisabolol oxides A (25.5% to 28.7%), and α-bisabolol oxides B (12.2% to 30.9%). The essential oil is well-known for its anti-inflammatory, antiseptic, and spasmolytic properties [73,74].

Specifically, in 2002, Hernández-Ceruelos et al. [75] conducted an investigation with the primary purpose of evaluating the chemoprotective capacity of a chamomile essential oil (CEO) on the sister chromatid exchanges (SCE) produced by daunorubicin and methyl methanesulfonate in mouse bone marrow cells. Initially, the authors obtained the CEO from flowers of *Chamomilla recutita* by steam distillation, and then they analyzed this oil by gas chromatography to identify the chemical species. Finally, 13 compounds were identified, including bisabolol and its oxides, β-farnesene, chamazulene, germacrene, and sesquiterpenes (Table 5).

Among the first studies that have shown the pharmacological activities of various components of the plant, the stand out compounds that have reported the anti-inflammatory capacity and the modulating effects of heat shock protein (Hsp) on apigenin and quercetin flavonoids, as well as the anti-inflammatory, antioxidant, and antiseptic activities detected in α-bisabolol, guaiazulene, and chamazulene [76,77].

**Antigenotoxic evidence for chamomile and its phytochemicals (α-bisabolol, apigenin, and chamazulene):** Despite chamomile tea being the most consumed herbal teas in the world, there is little research that confirms its antigenotoxic capacity [78,79].The first study was conducted by Stavric et al. (1996), who analyzed and compared the inhibitory effect of aqueous extracts of eight brands of common teas (derived from *Camellia sinensis*) and infusions of six randomly selected herbal teas on the mutagenicity of eight heterocyclic aromatic amines (HAA) using the Ames Salmonella typhimurium TA98 and S-9 assay. The majority of the extracts (in concentrations equivalent to 50 mg of tea leaves/plate) showed potent antimutagenic effects against HAA; even decaffeinated tea produced the same effect as that observed on “regular” teas. However, only some of them potentiated the mutagenicity of 3-amino-1-methyl-5H-pyrido [4,3-b] indole (Trp-P-2) and 2-amino-3,4,7,8-tetramethyl-3H-imidazo [4,5-f] quinoxaline (4,7,8-TriMeIQx) [80].

Possibly, this result motivated Hernández-Ceruelos et al. [75,81] to evaluate the protective effect of chamomile essential oil (CEO) on sister chromatid exchanges (SCE) produced by daunorubicin (DAU) and methyl methanesulfonate (MMS) in bone marrow cells and germ cells. Their results showed that: (a) In the first experiment, the DAU did not produce alterations in the kinetics of cell proliferation, but a reduction in the mitotic index. Unlike the MMS that did not show any alteration of these parameters, (b) both studies confirmed that CEO is not a genotoxic agent; on the contrary, it has a dose-dependent inhibitory effect (5 mg/kg, 50 mg/kg, and 500 mg/kg) on the rate of SCE (both in somatic cells and in spermatogonia) induced by both mutagens, and (c) that the mechanism of action of the CEO waspossibly related to its antioxidant capacity, which is very similar to that observed with vitamin E [75,81].

Recently, and considering that some compounds present in the diet can alleviate excessive inflammation, which is a factor in the pathogenesis of common diseases, such as rheumatoid arthritis, atherosclerosis, and diabetes, three European herbs (*Matricaria chamomilla*, *Filipendula ulmaria* L., and *Salix alba* L.) were analyzed to treat inflammation and its protective effect against oxidative damage induced in an inflammatory process. Aqueous herbal extracts and isolated polyphenolic compounds (apigenin and quercetin) were incubated with THP1 macrophages to quantify interleukin-6 (IL-6) and tumor necrosis factor-alpha (TNF-α). The results showed that chamomile was the extract of lower anti-inflammatory potential and that both phenols significantly reduced IL-6 and TNF-α. Likewise, the comet assay confirmed that both phenols and extracts showed a protective effect against the oxidative damage generated by inflammation [82].

As mentioned, chamomile is a relevant source of natural products; however, the most studied phytochemicals to evaluate their antigenotoxic capacity are α-bisabolol (BISA) and apigenin (APG).

In this sense, only two studies have been carried out; in the first, the mutagenicity and antimutagenicity of BISA were analyzed in the Salmonella/microsome assay. The mutagenicity of BISA was evaluated in TA100, TA98, TA97a, and TA1535 *Salmonella typhimurium* strains, without and with the addition of S9 mixture. No increase in the number of revertant colonies over the negative (solvent) control values was observed with any of the four tester strains. Its antimutagenic capacity was tested by using a high non-toxic dose (150 micrograms/plate) against the direct action agents (sodium azide (SA), 4-nitroquinoline-N-oxide (4-NQNO), 2-nitrofluorene (2-NF), and nitro-o-phenylenediamine (NPD)) and indirect action agents (cyclophosphamide (CP), benzo [a] pyrene (B[a]P), aflatoxin B_1_ (AFB_1_), 2-aminoanthracene (2-AA), and 2-aminofluorene (2-AF)).In summary, BISA did not alter the mutagenic activity of SA and NPD, and showed only a weak inhibitory effect on the mutagenicity induced by 4-NQNO and 2-NF. The mutagenic effects of AFB_1_, CP, B[a]P, 2-AA, and 2-AF were all markedly reduced. These results suggest that BISA-induced antimutagenicity could be altered by an inhibitory effect on the metabolic activation of these promutagens [83].

The second study, developed by Anter et al. [84], evaluated the antigenotoxic effect of APG and BISA against the hydrogen peroxide using the wing spot test of *Drosophila melanogaster*. All the concentrations used showed a significant genoprotective effect and also induced apoptosis in HL-60 leukemia cells. This study suggests that the antioxidant activity of these phenols could be partially responsible for their beneficial activity [84]. With respect to Chamazulene, there is no scientific evidence that confirm this chemopreventive potential, although possibly, by its chemical structure, this property could be favored. In this sense, it has been suggested that this sesquiterpene possesses anti-inflammatory activity [85] and that it is involved in the radical scavenging activity [86].

### 2.4. Cocoa (Theobroma cacao L.)

**Overview:** Cocoa (also known as “food of Gods”) is an important agricultural product and the main raw material in chocolate manufacturing. It is obtained from beans of the cacao tree (*Theobroma cacao* L.) belonging to the Malvaceae family. This is a small, evergreen tree, native to tropical regions of the Americas [87]. The fruit is a squash-like pod that grows proximal to the trunk and to thicker branches, and each cocoa pod contains around 35 beans to 50 beans embedded in a mucilaginous pulp [88,89].

*Theobroma cacao* L. is commercially cultivated, mainly in West Africa (70%), followed by Asia, Oceania (15.6%), and Latin America (14.1%). World leaders in cocoa bean production are the Ivory Coast, Ghana, Indonesia, Nigeria, Cameroon, Brazil, Ecuador, the Dominican Republic, and Malaysia, supplying 90% of the world production. In recent years, the world demand for cocoa products, such as chocolate, has significantly increased. The popularity of these products is related to their unique sensory and pleasant melt-in-the-mouth characteristics; but particularly for the scientific evidence that supports its health benefits and its role as a possible functional food. The main varieties of *Theobroma cacao* L. that are commercially exploited to make cocoa and chocolate are *Forastero, Criollo, Trinitario*, and *Nacional* [88].

The healing and medicinal benefits of cacao were appreciated by the ancient Mayan and Aztec civilizations. Such appreciation has currently increased, mainly for its anti-inflammatory, anti-allergenic, anti-carcinogenic, and antioxidant qualities. In the last decades, new properties, such as the ability to alleviate high blood pressure, and to control cholesterol, obesity, constipation, diabetes, bronchial asthma, chronic fatigue syndrome, and some neurodegenerative diseases have been discovered. It also helps improve cardiovascular health and emotional states, and exerts protective effects against neurotoxicity [90].

Various chemical components from raw cacao beans participate in the formation of specific cocoa flavors due to certain changes occurring throughout the process. These components are alkaloids (methylxanthines), polyphenols, proteins, and carbohydrates (Figure 2). Theobromine (3,7-dimethylxanthine) is the major alkaloid of cacao. The seeds have three main groups of polyphenols: Catechins or flavan-3-ols (ca. 37%), anthocyanins (ca. 4%), and proanthocyanidins (ca. 58%) [88,91,92,93].

**Figure 3:**
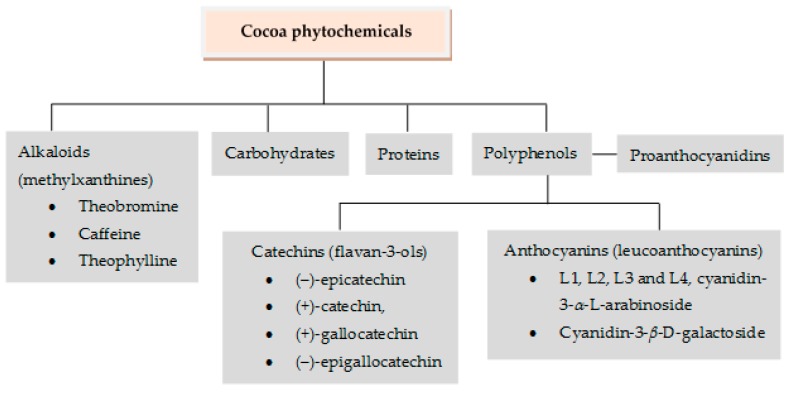
Main phytochemicals of cocoa.

**Antigenotoxic evidence for cocoa and its phytochemicals (theobromine, epicatechin, epigallocatechin, and catechin):** Although cacao products, such as chocolate, are commonly consumed for pure pleasure and chocolate is the main source of cocoa, which contains a largeamount of polyphenols (Figure 3), cacao is not among the main sources of polyphenols consumed by humans. In fact, there are data indicating that this agricultural product represents less than 10% of polyphenols’ intake. This same tendency is observed in the studies focused on its antigenotoxic potential, which started in this century. In 2001, the anticlastogenic effect of cacao liquor extract (CLE) against the formation of micronuclei induced by mitomycin C (MMC) in bone marrow cells and peripheral blood cells of mice was investigated. The results were that the frequency of both types of micronucleated cells was significantly reduced when the CLE was orally administered to the animals 6 h before the intraperitoneal injection of MMC. Subsequently, in 2016, the inhibitory potential of cacao (roasted and unroasted) against the genotoxicity induced by tetracycline in Swiss albino mice were both evaluated and compared. Results showed that instant cacao had the highest antigenotoxic potential to reduce micronucleated polychromatic erythrocyte formation induced by tetracycline. Both findings suggested that cacao is significantly effective in micronuclei reduction and damage prevention to DNA. The same study also showed that one of the mechanisms of action could include the elimination of reactive oxygen species (ROS) generated by MMC and tetracycline [94,95].

Considering that cocoa contains antioxidants that might inhibit the harmful effects of ROS, the effect of cocoa extract (CoE) consumption integrated as a bioactive compound into ready-to-eat meals on oxidative stress at the level of DNA in overweight/obese subjects was analyzed. Fifty volunteers participated in a four-week double-blind, randomised, placebo-controlled parallel nutritional intervention. Half of the volunteers received meals supplemented with 1.4 g/day cocoa extract, while the other half received control meals, both within a 15% energy restriction diet. Using lymphocytes from both groups, the endogenous strand breaks, oxidised bases, and resistance to H_2_O_2_-induced damage were also quantified by the comet assay. The lymphocytes of individuals treated with CoE did not show relevant changes in the oxidative state of DNA. However, the samples from both groups when compared showed a decrease in oxidized bases. Therefore, the subjects who started the intervention with higher levels of damage showed a greater reduction in the oxidized bases after four weeks in comparison to those who had lower initial levels; which suggests that a better protective effect could be present for a longer period of time [96].

As mentioned, cocoa is rich in procyanidins, and theobromine is the most available in human plasma, followed by caffeine, (−)-epicatechin, and (+)-catechin. However, after conducting a literature search, no evidence of the antigenotoxic potential of theobromine was found. With respect to caffeine, different investigations have confirmed this characteristic, but in most studies, caffeine extracted from coffee has been used, so it will not be considered in this review.

Therefore, the antigenotoxic evidence analysis will be focused on its main catechins ((−)-epicatechin, (+)-catechin, and (−)-epigallocatechin). For the evaluation of these flavonoid phenols, three experimental models have been employed (sister chromatid exchange, micronucleus assay, and single cell electrophoresis). In the first one, the protective effect of (+)-catechin and (−)-epigallocatechin gallate (both extracted from green tea) was evaluated against the damage produced by four trihalomethanes (chloroform (CHCl_3_), dichlorobromomethane (CHCl_2_Br), dibromochloromethane (CHClBr_2_), and bromoform (CHBr_3_)) and paraquat (PQ) in two different types of cell cultures. In summary, the results showed that the addition of crude catechin to the SCE assay system mainly suppressed the ability of CHCl_3_ and CHBr_3_ to induce SCEs in rat erythroblastic leukemia cells and that this reduction depended on the crude catechin dose. Likewise, both polyphenols in concentrations above 1.0 microM were able to decrease the frequencies of SCEs induced by PQ in these cells [97,98].

Law et al. [99] studied alpha-particle induced and medium-mediated bystander effects in Chinese hamster ovary (CHO) cells through micronucleus (MN) assay. They showed that signal transduction from irradiated cells to bystander cells occur within a short time after irradiation. They also evaluated the effects of ROS (reactive oxygen species)-scavenging catechins in the medium before irradiation. At the end of the study, they observed decreases in the percentage of bystander cells with MN formation and thus proved the protection effect of catechins on bystander cells from radiation [99]. The comet assay has been the most used technique to evaluate the antigenotoxic potential of these flavonoid phenols. Table 6 summarizes and analyzes the most relevant data of these studies.

### 2.5. Ginger (Zingiber Officinale)

**Overview:** Ginger (*Zingiber officinale*) is a member of the Zingiberaceae family of plants. The plant is native to Asia, but is now cultivated in the West Indies, Africa, India, and some tropical regions of America (Southern Mexico, Honduras, Nicaragua, Costa Rica, Panama, Colombia, Venezuela, Northern Brazil, Ecuador, and Bolivia). The underground stem “rhizome” (often called “ginger root”) is processed into a powder, syrup, volatile oil, and an oleoresin [105,106]. Its color varies from white to brown depending on whether the external cover is scraped off and the way it was initially treated. Its use in culinary applications dates as far back as the 13th century. Among all spices, it exhibits one of the greatest diversity of uses, such as in dietary supplements, beverages (e.g., ginger ales), and food products (curry powder, confectionaries, soups, jams, and baked goods). In addition to its culinary fusion, it has been used for a wide variety of health disorders, such as colds, fever, arthritis, stomach upset, asthma, diabetes, digestive problems (nausea and vomiting), menstrual irregularities, and as an appetite stimulant [105,106].

The rhizome contains fats (3%–6%), carbohydrates (60%–70%), protein (9%), crude fiber (3%–8%), water, and volatile oil (2%–3%). The characteristic flavor of ginger is due to zingerone, shogaols, gingerols, and volatile (essential) oils that comprise up to 3% of ginger on a fresh weight basis. The quality and quantity of biologically active constituents of ginger depend on its cultivation practices and postharvest treatment. The chemical components of the ginger rhizome can vary considerably, depending on the location of cultivation and whether the product is fresh, dried, or processed. The pungency of fresh ginger results from a group of phenols, the gingerols, of which [6]-gingerol (1-[40-hydroxy-30-ethoxyphenyl]-5-hydroxy-3-decanone) is the most abundant. Shogaols, which are dehydrated forms of gingerols resulting from thermal processing, also give dry ginger a pungent flavor. Fresh ginger may contain a 5-deoxy derivative of ginger called paradol. Ginger contains about 1% to 3% volatile oil that imparts a distinctive odor to ginger and which is composed mainly of monoterpenoids and sesquiterpenoids, including camphene, borneol, zingiberene, sesquiphellandrene, and bisabolene. All phytochemicals together contribute to its beneficial effects on health [106,107].

**Antigenotoxic evidence for ginger and its phytochemical (Gingerol):** Despite the common and local consumption of ginger as a food condiment, some studies analyze its protective effects against the damage produced by genotoxic substances. The main evidence that suggests its antigenotoxic potential has emerged due to its antioxidant capacity. One of the first studies was the investigation performed by Odunola (2003) [108], who evaluated the modulatory effect of the aqueous extracts of some food condiments (garlic, ginger, sconio, and cloves) on the clastogenic effects of sodium arsenite in mouse bone marrow cells using the micronucleus assay. The results were brief and confirmed that a pre-treatment of mice for seven days with extracts of the condiments orally administered before the oral dose of sodium arsenite (2.5 mg/kg) markedly reduced the number of MNPE of the bone marrow. The degree of reduction of the clastogenic effect of arsenite was as follows: Ginger > garlic > cloves > sconio. His conclusion was that this reduction of arsenite-induced clastogenicity by aqueous extracts of the condiments might be partially due to the antioxidant properties of their chemical constituents. Later, Bidinotto et al. [109] evaluated the chemoprotective effect of a ginger extract (GE) on the DNA damage induced by N-butyl-N-(4-hydroxibutyl) nitrosamine (BBN)/N-methyl-N-nitrosourea (MNU) in Swiss mice using the comet assay as well as the same technique that was used in the previous study.The animals were fed for 18 weeks with diets containing ginger extract (1% and 2%) and applied four intraperitoneal injections of MNU and a continuous treatment of BBN. The results were that the GE is not a genotoxic agent and does not reduce or alter the levels of DNA damage induced by the BBN/MNU treatment during the exposure.

The comparison of these studies, whose results were contradictory, was possibly the reason why Jayakumar and Kanthimathi (2012) [110] explored the protective effect of nine dietary spices against DNA damage induced by H_2_O_2_ and nicotine. Murine fibroblast cells (3T3-L1) and human breast cancer (MCF-7) were pretreated with spice extracts and then exposed to both genotoxic agents. Using the comet assay, it was evidenced that the concentration of the ginger (50g/mL) decreased 68% of the DNA damage of the 3T3-L1 cells produced by H_2_O_2_. The pre-treatment with ginger confirmed a relevant reduction on nicotine-induced cancer cell migration. These results confirmed that ginger is the best spice with this genoprotective potential and that a possible mechanism of action is related to its antioxidant capacity [110].

Recently, the anticlastogenic effect of ginger essential oil (GEO) on the antioxidant status and chromosomal damage in mice exposed to gamma irradiation was studied by means of the micronucleus assay, chromosomal aberration, and unicellular electrophoresis analysis. The results confirmed that GEO significantly decreased the frequency of micronuclei, inhibited the formation of chromosomal aberrations, and protected against cellular DNA damage in bone marrow cells as revealed by the comet assay. These data support the use of GEO as a compound with radioprotective potential, whose possible mechanism of action is related to its antioxidant property [111].

With respect to 6-Gingerol (6-G), the main component of ginger, only three in vitro studies have been carried out to evaluate its antigenotoxic potential. The first one evaluated the chemoprotective effect of 6-G against patulin (PAT)-induced genotoxicity in HepG2 cells. Both the comet assay and the micronucleus test were used to evaluate this possible property; likewise, for further exploration of the underlying mechanisms of action, the intracellular generation of reactive oxygen species (ROS) and the level of reduced glutathione (GSH) were evaluated. The results showed that 6-G significantly reduced DNA strand breaks and micronucleus formation caused by mycotoxin. In addition, the pretreatment with 6-G effectively suppressed the formation of intracellular ROS and attenuated the GSH depletion induced by PAT in this cell type. These data are evidence that the antioxidant activity of 6-G might play an important role in its antigenotoxic capacity [112].

Subsequently, Meschini et al. [113] evaluated the protective capacity of 6-G on the clastogenicity of N-methyl-N’-nitro-N-nitrosoguanidine (MNNG) and 7,12-dimethylbenz (α) anthracene (DMBA) in HepG2 cells. After carrying out pre-treatments with 6-G and combined treatments (6-G plus each mutagen), they found that this phytochemical significantly reduced the frequency of MN induced by MNNG and DMBA [113].

In the last research (2017), they observed that 6-G also significantly reduced DNA strand breaks caused by mono (2-ethylhexyl) phthalate (MEHP); a metabolite of di (2-etylhexyl) phthalate that is widely used as a plasticizer in medical devices. In this case, the comet assay was the technique used to monitor breaks in the DNA chain induced by MEHP in endothelial cells of the human umbilical vein (HUVEC), as well as the quantification of malondialdehyde, glutathione, and superoxide dismutase to explore its possible mechanism of action. In this sense, 6-G decreased the levels of malondialdehyde and increased the level of glutathione and the activity of superoxide dismutase, whereby, joining these results with those obtained in the previous studies, strongly indicate that the mechanism may be related to an antioxidant activity [114].

### 2.6. Bay Laurel (Laurus nobilis L.)

**Overview:***Laurus nobilis* L. is a member of the family, Lauraceae, that comprises 32 genera and about 2000–2500 species. Laurus is also known as sweet bay, Grecian laurel, true bay, bay tree, and bay laurel. Its natural habitat is the tropical and sub-tropical Himalayas areas. Turkey, Algeria, France, Greece, Morocco, Portugal, Spain, Belgium, Mexico, Central America, and the Southern United States are the main commercial production centres of bay laurel [115,116].

Bay laurel is an evergreen tree that grows to 2 m–5 m and under very favorable conditions can reach up to 20 m. It has a smooth and reddish-brown bark. Its leaves are lanceolate and leathery with shiny upper sides with a pleasant smell and a bitter taste. The fruit is a drupe the size of a small grape containing one seed. It is black-blue when ripe and has a sharp flavor. Generally, its leaves and the essential oil are the most utilized parts of the plant for its aromatic qualities. The fruit is rarely used due to its bitter taste [117,118]. In ancient times and different cultures, it was believed that the bay laurel tree held magical powers, warding off evil witchcraft and disease. The Greeks considered it as a medicine that could protect against diseases, especially against the plague. Bay laurel was not only used in ceremonies and rituals in the past, but also as a herbal medicine. Hippocrates used all parts of the plant as a remedy for a variety of ailments, both internal and external. Nowadays, laurel is mostly used as a pain remedy and against ailments related to the upper part of the digestive tract. It is also used for flu, bronchitis, and to stimulate appetite by increasing the secretion of digestive fluids. Likewise, both the leaves and their extract have been used as compresses for sprains, as a remedy for dandruff, and as an insect repellent agent. Regarding bay laurel essential oil, it has been used in aromatherapy, often combined with other essential oils (such as coriander, eucalyptus, ginger, juniper, lavender, rose, rosemary, and thyme), and as an ingredient in commercial products, such as cosmetics, toiletries, and perfumes, particularly men’s aftershaving lotions and foams [117,118].

Currently, bay laurel essential oil has been shown to possess many interesting properties (e.g., nematicidal, insecticidal, antifungal, acaricidal, anticonvulsant, antiseptic, antidiarrheal, antimycotic, antimicrobial, anti-inflammatory, anticarcinogenic, and antioxidant), that is to say, it has a wide spectrum of applications in many fields, including the health and food industry (culinary uses) [117]. In the food industry, laurus (both leaves and the essential oil) is widely used as a spice and condiment. The dried leaves are used as a seasoning for pies, soups, sauces, marinades, stews, and pickles. The leaves are used as an ingredient in many spice mixtures, like the famous French “bouquet garnii” [117,119,120].

**Antigenotoxic evidence for laurel and its phytochemicals:** The bay laurel has not been a plant of great scientific interest in relation to its chemopreventive aspect, especially in research aimed at evaluating its antigenotoxic capacity. Like other plants, there is little scientific evidence in the literature about this latter capacity and most of this evidence has been developed in in vitrotests. These studies began in 2011 when the efficacy of a laurel leaf extract (LE) against the toxicity of 2,3,7,8-tetrachlorodibenzo-p-dioxin (TCDD) in a primary culture of rat hepatocytes was tested by means of a micronucleus assay. The extract (concentrations of 50 mg/L, 100 mg/L, and 200 mg/L) was added to a culture with TCDD for 48 h. Subsequently, the oxidative damage was evaluated by measuring the total antioxidant capacity (TAC) and the total oxidative stress (TOS); as well as the DNA damage using the test mentioned before. The resuts were that the group of hepatocytes cultured with TCDD decreased the TAC and significantly increased the TOS and the frequency of micronucleated cells. In contrast, those cultures exposed to the combination of extract plus TCDD, where the levels of TOS did not change, the TAC increased significantly and the LE counteracted the toxic effects induced by TCDD in a dose-dependent manner [121].

Later, Türkez and Toğar (2013) conducted two studies to evaluate the effect of *Laurus nobilis* leaf extract (LNE) against the genetic and oxidative damage induced by aluminum phosphide (AlP), a colorless and flammable pesticide that is commonly used to control insects, nematodes, weeds, and pathogens in crops, forests, ornamental nurseries, and wood products. Initially, the frequency of sister chromatid exchange (SCE) and chromosomal aberrations (CA) were quantified in cultured human blood cells in the presence of a metabolic activator (S9 mix). Subsequently, the frequency of micronuclei, as well as the total antioxidant capacity and total oxidative status levels, were determined in rats treated with both compounds for 14 days. Both investigations showed that: (a) The pesticide increased the number of SCE and CA compared to the control group, while the combined application of LNE (25 mg/L, 50 mg/L, 100 mg/L, and 200 mg/L) plus AlP induced a significant decrease in the rates of these parameters. (b) AlP also increased the frequency of MN and altered the levels of TAC and TOS, and (c) this damage was significantly reduced by combining both compounds, since LNE reduced the oxidative stress and suppressed the genetic damage induced by AlP in bone marrow cells in vivo. In conclusion, the results suggested that the protective effect of LNE could be attributed to the laurel’s ability to eliminate free radicals [122,123].

Several studies have shown the chemical composition of different parts of the herb (leaves, flowers, stems, bark, or fruits) and its essential oil. In general, the leaves contain sesquiterpenes, lignan glycosides, alkaloids, mucins, bitter substances, tannins, and resins. While the main ingredients of laurus essential oil are eucalyptol, eugenol, linalool, geraniol, and alpha-pinene [117,118,120,124]. Unfortunately, there are no studies where the antigenotoxicity of these compounds extracted directly from the laurel has been evaluated. Thus, Table 7 summarizes and analyzes some studies that suggest the genoprotective capacity of the main phytochemicals of the essential oil. However, only the most relevant research developed during the last years is included.

### 2.7. Marigold (Calendula Officinalis Linn.)

**Overview:** The genus, Calendula (Asteraceae), is native to the Mediterranean countries and includes approximately 25 herbaceous annual or perennial species; the most common are *Calendula officinalis* Linn., *Calendula arvensis* Linn., *C. suffruticosa* Vahl., *C. stellata* Cav., *C. alata* Rech., and *C. tripterocarpa* Rupr. [134,135].

Specifically, *Calendula officinalis* (also called “pot marigold”) is an annual or biennial plant that is grown all over the world. It is a hairy grass 30 cm to 60 cm tall, with simple, thick, and remotely denticulated leaves. Its flowers can be bright yellow to orange and are used both ornamentally and for the preparation of pharmaceutical and cosmetic products [135,136].

Marigold has been traditionally used in the treatment of inflammations of internal organs, gastrointestinal ulcers, and dysmenorrhea, and as a diuretic and diaphoretic in convulsions. It is also used for inflammations of the oral and pharyngeal mucosa, wounds, and burns. Calendula is a cleansing and detoxifying herb and the infusion is used for chronic infection treatments. The dried flower heads have been used for their antipyretic, anti-tumor, and cicatrizing effects. The infusion of these flowers applied topically works as an antifungal and antiseptic in wounds, marks, freckles, sprain, and conjunctivitis. Calendula infusion is also used for eyewashes and gargles, and to cure diaper rashes and other inflammatory conditions of the skin and mucous membranes. In general, pharmacological studies agree that both the leaves and the fruits have antibacterial, antiviral, antipyretic, anti-inflammatory, antiepileptic, anti-tumor, and antioxidant properties. Most of the studies that have partially investigated the phytochemicals of *Calendula officinalis* indicate that they belong to a number of chemical families, most notably the polysaccharides, hemicelluloses, carotenoids, flavonoids, triterpenes, saponins, phenolic acids, tannins, coumarins, mucilage, xanthophylls, phytosterols, resins, and essential oil. All these potentially active chemical constituents justify the diversity of pharmacological applications of the plant [135,136,140,141].

**Antigenotoxic evidence for marigold and its phytochemicals:** Despite presenting a wide range of pharmacological actions related to the phytochemicals of their chemical composition, just as laurel, marigold has not fully awakened the scientific interest to confirm its genoprotective potential. There are only two in vivo studies in which the antigenotoxic capacity of extracts of *C. officinalis* against the action of some mutagens has been analyzed so far. In the first one, Dimer-Leffa et al. [142] investigated the genotoxicity/antigenotoxicity of two types of extracts of *C. officinalis* against the damage produced by MMS. Male CF-1 mice treated with ethanolic (250 mg/kg or 500 mg/kg) or aqueous (90 mg/kg) extracts of *C. officinalis* for two weeks prior to the administration of MMS showed that no mutagenic effect was induced with any dose of both extracts in blood and bone marrow samples from animals analyzed by the comet assay and the micronucleus test, respectively. In contrast, the unicellular electrophoresis showed a protective effect of both types of extracts, repairing the DNA damage caused by MMS; however, with the MN test, only the aqueous extract revealed this same genoprotective effect [142].

In relation to the second study, male Sprague-Dawley rats were used to evaluate the hepatoprotective effect of a calendula flower extract on oxidative stress and aflatoxins (AFs)-induced genotoxicity by the micronucleus assay. The animals treated with the extract for one week before the AFs treatment showed a significant decrease in oxidative damage markers, in the number of micronucleated cells, and DNA fragmentation [143]. The results of both studies suggest that calendula extracts have antigenotoxic effects for their high content of phenolic compounds. Some studies have shown that carotenoids and flavonoids are the bioactive compounds commonly found in the flowers at 0.078% and 0.88% respectively. Among the carotenoids are α, β, and γ-carotene, violaxanthin, rubixanthin, citroxanthin, flavoxanthin, galenin, lycopene, valentiaxanthin, auroxanthin, microxanthin, β-zeacarotene, mutatoxanthin, lutein epoxide, and lutein; while the most common flavonoids are isorhamnetin 3-0-glycoside, isorhamnetin, rutinoside, quercetin, calendofloside, calendoflavoside, calendoflavobioside, isoquercetin, quercetin, rutoside, and kaemferol [143].

Other compounds identified are: (a) Triterpenes (such as 3,16,21 trihydroxy-ursaeno, ursadiol, heliantrioles (A_0_ B_1_, B_2_ and C), loliolide, 3,16,28 trihydroxy olean-12-ene, 3,16,28 trihydroxy lup-20 ene, and calenduloside F); (b) essential oil (conformed basically by depedunculatin, α and β ionones, oxido-transcarophyllene, carvone, caryophyllene, cardinoles, geranyl acetone, oplopanone, γ-mourolene, α-cardinone, and torryol); (c) phenolic acids (mainly coumaric, gentisic, vanillic, caffeic, o-hydroxyphenylacetic, protocatechinic, ferulic, p-hydroxybenzoic, and salicylic); (d) coumarins (such as scopoletin, umbeliferone, and esculetin); and (e) saponins [135,136,140,141,144].

In 2006, lutein from marigold flowers was extracted and purified with the purpose of evaluating its antimutagenic effect through the Ames test (in the presence and absence of S9), as well as its anticlastogenic potential using the chromosomal aberration test in cells taken from a Chinese hamster ovary. In addition, its antioxidant activity was analyzed by means of the photo chemiluminescence (PCL) assay. The results were that lutein showed greater antioxidant activity than β-carotene and lycopene. In the same way, there were no mutagenic events in any of the doses evaluated (334 micrograms/plate, 668 micrograms/plate, and 1335 micrograms/plate); on the contrary, it presented a dose-dependent antimutagenic effect against mitomycin C, 2-aminofluorene, and cyclophosphamide. A similar phenomenon was observed with the chromosomal aberration test, confirming its anticlastogenic capacity in the three concentrations used (66.8 mg/L, 133.5 mg/L and 267.0 mg/L) [145].

### 2.8. Roselle (Hibiscus Sabdariffa Linn.)

**Overview:** The genus, Hibiscus (Malvaceae), includes more than 300 species of annual or perennial herbs, shrubs, or trees. However, the best-known species that have been used and studied is *Hibiscus sabdariffa* Linn (Hs), which has different common names depending on the country or place of use. For example, in England, it is commonly known as roselle, karkadé (in France, Egypt, Arabia, Sudan, and the North of Africa), bissap (Senegal), omutete (Namibia), gongura or lalamgari (India), and jamaica in Latin America [146,147]. According to the nature of its production, Hs exists in two varieties; those that produce a lot of fiber (altissima and bhagalpuriensis) and intermediate varieties of fiber and calyces (intermedius, albus, and ruber) [147,148,149].

Although its origin and native distribution is uncertain, some authors believe it was in India or Saudi Arabia, while others have suggested that Hs was domesticated by the black populations of Western Sudan (Africa) sometime before 4000 BC. Nowadays, it is widely cultivated in both tropical and subtropical regions, including India, Saudi Arabia, China, Malaysia, Indonesia, the Philippines, Vietnam, Sudan, Egypt, Nigeria, and México [147,148,150].

Because its cultivation is relatively easy, roselle has been used as an ornamental plant, and in the food and pharmaceutical industries. In the culinary field, their fresh or dried calyces are used in the preparation of herbal drinks, hot and cold beverages, fermented drinks, wine, jam, jellied confectionaries, ice cream, chocolates, flavouring agents, puddings, and cakes. With respect to its medicinal uses, in India, Africa, and Mexico, infusions of the leaves are traditionally used for their diuretic, cholerectic, febrifugal, and hypotensive effects, decreasing the blood viscosity and stimulating intestinal peristalsis. In other countries, it has been used to treat heart and nerve diseases, to help reduce the body temperature, to control pain and coughs, and to decrease some genital problems [148,150,151,152,153].

In general, the leaves and extracts of roselle have shown antibacterial, antifungal, antiparasitic, antipyretic, antinociceptic, anti-inflammatory, anti-obesity, antidiabetic, antihypertensive, anti-anemic, anti-cholesterol, diuretic, hepatoprotective, nephroprotective, and antioxidant effects [148,150,154,155].

The nutritional composition of the fresh or dried calyces of *H. sabdariffa* (cHs) differs among the different genetic varieties of the plant and the environmental conditions of its harvest. In general, the studies indicate that the leaves and cHs contains protein, fat, carbohydrates, ascorbic acid, β-carotene, calcium, iron, and fibre. With respect to the main bioactive constituents of *Hibiscus sabdariffa* Linn. relevant in the context of their pharmacological effects, are the organic acids (such as citric, hydroxycitric, hibiscus, malic, tartaric, oxalic, and ascorbic), anthocyanins (among the most important are delphinidin, delphinidin-3-sambubioside (hibiscin), cyanidin-3-sambubioside (gossypicyanin), and cyanidin-3,5-diglucoside), and flavonoids (mainly, hibiscitrin (hibiscetin-3-glucoside), sabdaritrin, gossypitrin, gossytrin, quercetin, and luteolin), chlorogenic acid, protocatechuic acid, pelargonidic acid, eugenol, and ergosterol] (Table 4) [147,148,149,154].

**Antigenotoxic evidence for roselle and its phytochemicals:** In recent decades, *Hibiscus sabdariffa* has received considerable attention in the nutritional field (especially its flower that is used in the preparation of fresh water, tea, desserts, and gelatins) and for its various medicinal uses. However, it has been the plant with the least studies devoted to its genoprotective potential. Practically, there are only three research studies in the literature that have evaluated extracts of the plant (ethanolic, methanolic, and aqueous), with the purpose of demonstrating its antigenotoxic capacity.

The first one was carried out in 2004 on the protective effect of an aqueous extract of *Hibiscus sabdariffa* against sodium arsenite-induced micronuclei formation in erythrocytes of albino mice bone marrow. Its anticlastogenic potential was evidenced by a significant dose-dependent reduction (50 mg/kg, 100 mg/kg, and 150 mg/kg) in the number of micronucleated, polychromatic erythrocytes (MNPE) induced by sodium arsenite [156].

Later, Farombi and Fakoya [157] investigated the antioxidant effect and the capacity of the elimination of free radicals of two fractions of the ethanolic extract (HSCF, fraction soluble in chloroform, and HSEA, fraction soluble in ethyl acetate) obtained from the dried flowers of *H. sabdariffa*; as well as the anticlastogenic potential of both fractions against sodium arsenite, using the same technique as in the previous study. The results showed that both HSCF and HSEA were better scavenger agents of O_2_, OH, and H_2_O_2_ in comparison to quercetin and alpha-tocopherol. Its antioxidant potential was 70% at concentrations of 380 microg/mL, 500 microg/mL, and 1000 microg/mL. On the other hand, rats pretreated with HSCF and HSEA significantly reduced the induction of MNPE by sodium arsenite after 24 h in 60% and 70%, respectively. These results suggested that extracts of *H. sabdariffa* have a strong antigenotoxic activity and a free radical scavenging potential on active oxygen species [157].

Using two different techniques to confirm the genoprotective action of plants of the genus, Hibiscus, Brazilian researchers determined the protective effect of a methanolic extract of *H. tiliaceus* L. in V79 cells against the cytotoxicity and genotoxicity induced by H_2_O_2_ and tert-butyl hydroperoxide (t-BHP). Those scientists evaluated the DNA damage with the comet assay and the micronucleus test in binucleated cells, as well as the degree of lipid peroxidation. They observed that in concentrations varying from 0.001 mg/mL to 0.1mg/mL of *H. tiliaceus* L. methanolic extract (HME), there was no cytotoxic, genotoxic, or mutagenic effect. They also confirmed that the pretreatment of HME was able to decrease the mutagenic effect and the increase in lipid peroxidation produced by these genotoxins [158].

Recently, Vilela et al. (2018) [159] analyzed the presence of phenolic compounds in extracts of *H. acetosella* leaves (EHa) by high-performance liquid chromatography (HPLC) and determined the antigenotoxic effect of these extracts against the damage produced by methyl methanesulfonate (MMS) in mice. The HPLC results showed the presence of caffeic acid, gallic acid, gallocatechin, coumaric acid, and 3,4-dihydroxybenzoic acid. On the other hand, using the micronucleus test and the comet assay, they showed that the animals treated with EHa (dose of 50 mg/kg or 100 mg/kg) plus the alkylating agent significantly decreased the DNA damage [159].

### 2.9. Rosemary (Rosmarinus Officinalis L.)

**Overview:** Rosemary (*Rosmarinus officinalis* L.) is a common domestic plant native to the Mediterranean region, whose name derives from the Greek *rhops* and *myrinos* that means “marine shrub” for it grows near the coast. It is usually found wild in rocky and sandy areas near the sea, but due to its adaptability, rosemary easily reproduces itself in different parts of the world. Currently, it is grown in Asia, Europe, and Latin America. In Mexico, it grows wild in the states of Guerrero, Hidalgo, Jalisco, Michoacan, Morelos, Oaxaca, Puebla, Sonora, Tlaxcala, and Veracruz [160,161,162,163,164]. Rosemary belongs to the family, Lamiaceae; it is a shrubby plant with prismatic stems, woody and branched. It is a perennial herb with fragrance, whose leaves are narrow, sharp, and small; the shape of bright green spikes with revolute margins; and woody and branched stems. The size varies from 0.5 m to 1 m in height, flowering twice a year in spring and autumn. The flowers have a characteristic light blue color with small violet spots [163,164].

Rosemary is a plant rich in different active ingredients that act on almost all organs of the body; for this reason, the International Herb Association named it Herb of the Year in 2000 [164]. Rosemary has a high content of volatile essential oil, whose active ingredients are flavonoids, phenolic acids, triterpene acids, triterpene alcohols, tannic acid, resin, and different bitter components, which together generate a tonic and stimulating action in the central nervous, cardiovascular, genitourinary, hepatic, reproductive, and respiratory systems. In general, it has been used as a choleretic, cholagogue, antispasmodic, diuretic, and emmenagogue agent [163,164,165,166].

The rosemary essential oil is the most studied component, and its chemical composition depends on the geographical location where the plant grows (that is, soil type, climate, and height above sea level).

In general, the bioactive molecules of the volatile oil are camphor, 1,8-cineol, pinene, borneol, camphene, limonene, verbenone, caryophyllene, myrcene, and triterpenes (such as betulin and α-amyrin).The rosemary essential oil is utilised in lotions for the treatment of ailments, such as arthritis, gout, muscular pain, neuralgia, and wounds [163,164,167,168,169,170].

Due to the diverse pharmacological actions and its culinary and ornamental uses, in recent years, its commercial demand has grown and, nowadays, it is used as an essential ingredient of several products available in both the food and pharmaceutical industries.

**Antigenotoxic evidence for rosemary and its phytochemicals:** All the previously mentioned facts have motivated the analysis and research of a large number of scientific contributions that provide extensive information on rosemary, beyond its culinary and ornamental uses. In order to confirm its genoprotector potential, Darina Slamenova and her team from the Institute of Cancer in the Slovak Republic, in 2002, started a research focused on the antigenotoxic evaluation (using single cell gel electrophoresis) of an ethanol extract of rosemary (EER) against oxidative DNA damage induced by H_2_O_2_- and visible light-excited Methylene Blue in colon cancer cells CaCo-2 and hamster lung cells V79. Their findings showed that EER reduced the genotoxic activity of both agents after a long-term (24 h; 0.3 μg/mL) or short-term (2 h; 30 μg/mL) pre-incubation of cells; they suggested that the extract evidences a protective effect against the oxidative damage to DNA because of both OH radicals and singlet oxygen scavenging (^1^O_2_) [171]. Some years later, Aherne et al. [172] explored the cytoprotective and genoprotective effects of some aqueous extracts of plants (rosemary (*Rosmarinus officinalis* L.), oregano (*Origanum vulgare* L.), sage (*Salvia officinalis* L.), and echinacea (*Echinacea purpurea* L.)) against the damage induced by H_2_O_2_ in Caco-2 cells. Once the cells were pre-treated with each extract for 24 h and exposed to the H_2_O_2_, the cellular viability by the neutral red uptake assay (NRUA) and DNA damage were assessed by the so-called comet assay. The results indicated that all extracts induced cell injury, with echinacea the least toxic; however, rosemary, sage, and oregano protected against H_2_O_2_-induced DNA damage (olive tail moment and percentage tail DNA were reduced) [172].

These results probably motivated Zegura et al. [173] to evaluate the antioxidant and antigenotoxic effects of rosemary extracts in Salmonella typhimurium TA98 and HepG2 cells; which confirmed water soluble AquaROX (®) 15 and soluble oil VivOX (®) 40 rosemary extracts as reducers of mutagenicity induced by 4-nitroquinoline-N-oxide (NQNO) and 2-amino-3-methyl-3H-imidazo [4,5-F]quinoline (IQ) in the reverse mutation assay with Salmonella typhimurium TA98. Applying the same technique as in the previous studies, both extracts proved protection of the DNA from oxidative damage induced by t-butyl hydroperoxide (t-BOOH), benzo(a)pyrene (BaP), and 2-amino-1-methyl-6-phenylimidazo[4,5-b]pyridine (PhIP) in HepG2 cells. However, the most efficient protection was VivOX against indirect carcinogens. These results suggest that the mechanism of action of the extracts is related to their antioxidant capacity [173].

The last two investigations, using the single cell gel electrophoresis, were developed in 2014. In the first one, two rosemary extracts were compared and evaluated on human lymphocyte DNA damage induced by H_2_O_2_. Lymphocytes isolated from blood samples belonging to healthy volunteers were incubated with aqueous and ethanol extract of rosemary (0.05 mg/mL, 0.1 mg/mL, 0.5 mg/mL, 1 mg/mL, and 2.5 mg/mL) and H_2_O_2_ for 30 min at 4 °C. The results demonstrated that only the ethanolic extract was able to inhibit the percentage of DNA damaged by approximately 4.5% at the concentrations tested [174]. With respect to the second study, Pérez-Sánchez et al. [175] employed a combination of rosemary and citrus bioflavonoid extracts to inhibit harmful UV effects on human HaCaT keratinocytes and in human volunteers after an oral intake. Survival of HaCaT cells after UVB radiation was higher in treatments using the combination of extracts than in those performed with individual extracts, indicating potential synergic effects. The combination of extracts also decreased UVB-induced intracellular radical oxygen species (ROS), prevented DNA damage in HaCaT cells, and reduced chromosomal aberrations in X-irradiated human lymphocytes [175].

Using the eye white/white+ (w/w+) somatic mutation and recombination test (SMART) assay of *Drosophila melanogaster*, other assays considered in the area of genetic toxicology (Table 2) to evaluate the genotoxic and/or antigenotoxic effects of plants, vegetables, fruits, and drugs, the genoprotective action of laurel (*Laurus nobilis*), rosemary (*Rosmarinus officinalis*), verbena (*Verbenatriphylla*), fenugreek (*Trigonella foenum-graecum*), and nutmeg (*Myristica fragrens*) were compared with methyl methanesulfonate (MMS). The results confirmed that MMS was a positive compound to induce high frequencies of spots in Drosophila larvae. In contrast, all the extracts diminished the spots induced by this mutagen. Another observation was that the greater antigenotoxic effects corresponded to the extracts of nutmeg and rosemary in approximately 50% [176].

With respect to the rosemary phytochemicals, two different investigations were carried out in 2010, with the main objective of evaluating their anticlastogenic potentials. The first corresponds to an in vivo study, where the carnosic acid (CA) presented this property against the DMBA-induced confirmed clastogenesis induction. In general, the oral pre-treatment of CA for five days to DMBA-treated hamsters significantly reduced the frequency of bone marrow micronucleated polychromatic erythrocytes (MNPE) and the chromosomal aberrations. The CA was confirmed to have an antioxidant capacity and an effect on the modulation of phase I and II detoxification enzymes, and mechanisms of action, which, combined, can play a relevant role for its chemopreventive potential [177].The last study was conducted under in vitro conditions and aimed to investigate the ability of rosmarinic acid (RA) to prevent chemically induced chromosome breakage or loss and primary DNA damage using the micronucleus and comet assays with V79 cells, respectively. In this case, doxorubicin (DXR, 0.5 microg/mL) was used as a genotoxic agent. The cultures were treated with different concentrations of RA (0.28 mm, 0.56 mm, and 1.12 mm) alone or in combination with DXR; the result was that RA exerted no genotoxic effect, but significantly reduced the frequency of micronuclei and the extent of DNA damage induced by DXR at the three concentrations tested. Again, these results suggest that the antioxidant activity of RA may be involved in the reduction of DNA damage [178].

## 3. Natural Resin

### 3.1. Propolis (Bee Glue)

**Overview:** Propolis is a resinous substance of natural origin, gathered by bees from different parts of plants, shoots, and exudates [179,180]. Bees utilize it as a sealant for their hives and to avoid the decomposition of creatures killed by the bees after hive invasions [180,181,182]. Chemically, propolis is a lipophilic material that is hard and fragile when cold, but soft, flexible, and very sticky when hot, hence the name bee glue. It has an agreeable aromatic odor, and it varies in color depending on its origin and maturation. The compounds identified in propolis derive from three sources: Plant exudate collected by bees; substances secreted by the metabolism of the bees; and materials that are introduced during the creation of the resin [180,183,184]. The ethanolic extract of propolis (known as propolis balsam) is the most common, but there are other solvents used to separate and identify many of its components. Among the types of chemical substances found in propolis are waxes, resins, balsams/balms, aromatic oils, pollen, flavonoids, terpenoids, and other organic materials (Table 8). The proportions of these substances are variable and depend on the place and time propolis is obtained [183]. Propolis has a long history of being utilized in popular medicine dating from at least 300 BC [180,183,184,185]; however, over the last decade, it has been found to possess diverse biological activities, and among the most prominent is its antioxidant, anti-inflammatory, antibiotic, and antifungal potential. Recently, propolis has gained popularity as a health beverage; thus, it has been widely utilized in foods and drinks, with the aim of preventing alterations of the heart and chronic degenerative diseases, such as diabetes and cancer [180,185,186,187].

**Antigenotoxic evidence for propolis and its phytochemicals:** There are different colors of propolis depending on the geographical area of origin and the type of plants or trees used for their extraction. The combination of colors varies from black, ochre, red, brown, light yellow, green, to a large number of brown tones; yellow and green being the most common. In the case of green propolis, this is produced by Brazilian bees from resins that they collect from the buds of the plant, “*Baccharis dracunculifolia*”. In general, all propolis have similar organoleptic characteristics; however, its quality may change slightly depending on the form of extraction. Due to its more than 300 components identified to date and its broad spectrum of biological activities, it has been considered a promoter agent for human health and an ideal candidate for possessing genoprotective properties.

Therefore, in the same way as broccoli, cocoa, and laurel, this natural resin has been the subject of study for several groups of researchers exploring its antigenotoxic potential. The evaluations on this property are relatively recent, starting in 2005, when the effect of an aqueous extract of propolis (AEP) on the formation of aberrant crypt foci (ACF) and DNA damage induced by 1,2-dimethylhydrazine (DMH) in the Wistar rat colon by comet assay was analyzed. AEP was orally administered at 0.01%, 0.03%, 0.1%, and 0.3% in the drinking water (ie, in approximate doses of 12, 34, 108, and 336 mg/kg body weight/day). The animals also received various injections of DMH and were sacrificed at different stages in order to evaluate the development of ACF in the distal colon and damage to the DNA. The results showed that the combined administration of AEP with DMH significantly reduced DNA damage induction in the distal colon. It was also observed that AEP had no effect on the formation of DMH-induced ACF in the rat colon [188].

The rest of the studies are listed in Table 9. In summary, three groups of researchers have made the greatest number of scientific contributions. The first group, from the Faculty of Medicine of Turkey, evaluated the protective effect of ethanolic extracts of Turkish propolis (EETP) against DNA damage induced by H_2_O_2_ [189] and γ-rays [190] in fibroblast cells using the comet assay. The results of both experiments showed a significant reduction of the damage generated by thegenotoxic agents in the cultures treated with the extract. Their findings suggest that the chemopreventive activity of EETP may occur under different mechanisms, including the antioxidant action.

The second group, with the greater number of studies carried out, has been directed by Benković V. and Oršolić N. Their contributions have revolved in evaluating the antigenotoxic, radioprotective, antitumor, and immunostimulatory potential of propolis in different presentations and administrations against diverse mutagenic and/or carcinogenic agents, using the comet assay, the micronucleus test, and the quantification of the number of chromosomal aberrations both in in vivo and in vitro conditions. The results of these contributions are as relevant as those from the previous group, because its protective effect was significant and comparable with other antioxidants, such as quercetin, naringin, and caffeic acid [191,192,193,194,195,196,197,198].

In the last group, Turkish researchers from the Atatürk University evaluated the effectiveness of this resin in modulating the aluminium chloride (AlCl(3)) induced genotoxicity and hepatotoxicity in the liver of rats (2010); and under in vitro conditions, analyzed this same property, but against 2,3,7,8-tetrachlorodibenzo-p-dioxin (TCDD)-induced toxicity in hepatocytes (2012). Using the micronucleus test in both studies, they found that the rats treated daily for 30 days with a combination of propolis (50 mg/kg) and AlCl(3) significantly reduced the number of micronucleated hepatocytes (MNHEPs). Similar results were observed when three concentrations of propolis (25 μM, 50 μM, and 100 μM) were added to plain culture or simultaneously with TCDD (5 μM and 10 μM) [199,200].

In relation to the propolis phytochemicals, different Brazilian researchers are the main authors who have generated scientific evidence about their antigenotoxic properties. The scientific group coordinated by Pollyanna Oliveira is the one that has carried out the largest number of studies since 2011. Initially, they explored the antigenotoxicity of baccharin both in vivo and in vitro using the micronucleus and comet assay. In the first case, they investigated the ability of this important constituent of *Baccharis dracunculifolia* to modulate the genotoxic effects induced by doxorubicin (DXR) and methyl methanesulphonate (MMS) in male Swiss mice; while in the other study, they analyzed the same property, but in V79 cells against MMS and H_2_O_2_. The results of both studies showed statistically significant differences in the DNA damage at the highest dose and concentration in comparison to the control group with the two tests used. With these results, baccharin can be considered a chemopreventive agent, whose possible antioxidant effect was responsible for reducing the genomic and chromosomal damages. Likewise, this chemical compound was also responsible for the antigenotoxicity also demonstrated by *Baccharis dracunculifolia*; the most important plant source of Brazilian green propolis [201,202,203].

Considering that baccharin is not the only phytochemical present in propolis, the same group of Oliveira (2013) [201] and the scientific group of de Azevedo Bentes Monteiro (2011) [204] explored the protective activity of artepillin C (3, 5-diprenyl p-coumaric acid-), one of the major phenolic compounds found in Brazilian green propolis. De Azevedo Bentes Monteiro Neto and colleagues analyzed the antigenotoxicity of artepillin C against the damage produced by DXR and MMS in male Swiss mice using the micronucleus and comet assays. Different doses of artepillin C (0.4 mg/kg, 0.8 mg/kg, and 1.6 mg/kg) were administered simultaneously with DXR (micronucleus test; 15 mg/kg) and MMS (comet assay; 40 mg/kg). The results showed that artepilin C itself was not genotoxic in any trial. In contrast, the number of micronucleated reticulocytes was significantly lower in the animals treated with artepillin C and DXR in comparison with the animals treated only with DXR. In the same way, the tested doses of artepillin C significantly reduced the extent of DNA damage in liver cells induced by MMS [204]. Unlike the previous study, de Oliveira et al. (2013) evaluated the protective effect of this phenolic compound, but under in vitro conditions using the same genetic assays.

Cultures of Chinese hamster lung fibroblasts (V79 cells) were treated with different concentrations of artepillin C (2.5 μM, 5.0 μM, 10.0 μM, and 20 μM) and combined with MMS (comet assay; 200 μM, and micronucleus assay; 400 μM). The study confirmed that all concentrations of artepillin C showed protective activity in relation to MMS-induced genotoxicity; a phenomenon that can be attributed to its antioxidant properties [201].

The last of the studies carried out so far belongs to Manzolli et al. (2015) [205], who evaluated the protective effect of chrysin (CR) against the oxidative damage produced by methylmercury (MeHg) in Wistar rats using the comet assay. Like the previous studies with baccharin and artepillin C, the results were quite favorable because the animals treated with the combination of CR and MeHg reduced the formation of comets in leukocytes and hepatocytes. In addition, the doses used of CR (0.10 mg/kg, 1.0 mg/kg, and 10 mg/kg) restored the glutathione levels. Taken together, all these findings indicate that the consumption of flavonoids, such as CR, baccharin, and artepillin C, can protect humans against the adverse effects of some genotoxins, including MeHg, MMS, and DXR [205].

### 3.2. Antigenotoxic Biomarkers in Cancer

The information on plants and vegetables compiled in the present manuscript demonstrates the importance of their genoprotector potential and confirms that this corresponds to a minimal amount of these, approximately only 30,000 species of edible plants throughout the world, where only about 150 species are cultivated and consumed in the human diet [38,209]. In the same manner, the impact is evidenced of these scientific assays (sister chromatid exchange, chromosomal aberrations, micronucleus, and comet assay) in the field of toxicological genetics for evaluating the genotoxic and antigenotoxic effect of these compounds and/or extracts deriving from plants, vegetables, and fruits, in preclinical as well as in clinical assays.

In this regard, it is convenient to consider that the investigations presented and their analyzed results with these antigenotoxic markers can be relevant in the prediction and/or the development of cancer, bearing in mind some important points:

1. To date, there is no exact cipher of the number of plants and fruits with antigenotoxic and anticarcinogenic properties. This cipher is related to diverse factors, including the fact that the investigators select the species to evaluate taking into consideration its use in traditional medicine or to a specific region, the presence of bioactive compounds of interest, and/or the amount of previous studies on the same species. In general, investigators have published the results of numerous species and/or families of plants, and their studies have included the complete plant, its extracts and juices, and some specific phytochemicals.

2. Different epidemiological data provide evidence that it is possible to prevent cancer and other chronic diseases, some of which share common pathogenic mechanisms, such as DNA damage, oxidative stress, and chronic inflammation. A complementary strategy is to render organisms more resistant to the mutagens/carcinogens and/or to inhibit the progression of the disease by means of the administration of chemopreventive agents. The latter can act in different stages, such as primary prevention, which is directed toward apparently healthy individuals, with the possibility of inhibiting mutation and the initiation of cancer by activating protector mechanisms in the extracellular as well as the intracellular environment. Also, with secondary prevention, when a premalignant lesion has been detected, and at a certain moment, it is possible to inhibit the progression of the tumor through these same extra- and intracellular mechanisms [7,210]. Therefore, the concordance issignificant between the results obtained in the genotoxic and/or antigenotoxic assays with respect to the presence of cancer, in which the majority of the studies have been conducted under experimental conditions. For example, Hung Kang et al. (2013) [211], employing the micronucleus and comet assay, found more than 90% agreement between invivo positive genotoxic assays with respect to more than 20 carcinogens classified by the International Agency for Research on Cancer (IARC). In addition, there are data that confirm that the comet assay has a high proportion of positive responses for genotoxic carcinogens in rodents [212].Similarly, the application has been suggested of at least three tests and/or the combination of different assays, in vivo as well as in vitro, to obtain reliable results [213], as in the case of Kirkland et al. (2005), who found 90% positive results with 553 carcinogens [214].

3. Finally, one must consider that all compounds, substances, or plant extracts that would be used in a clinical study must be previously evaluated in different investigation phases, with the purpose of confirming their pharmacological and/or therapeutic results, but especially, their toxic capacity. Thus, the route for the agents analyzed in the clinical prevention of cancer is long, and generally begins with the antigenotoxic identification of the candidate agent, followed by the evaluation of its potency by means of diverse tests and with the analysis of its mechanism and/or mechanisms of action. Once this is confirmed, it will be possible to evaluate its properties as a chemopreventive agent in phase, I, II, and III clinical assays to reduce the incidence of cancer. Among the main characteristics of the agents tested with chemopreventive potential is that of their preferably non-toxic, effective at low doses, low-cost, and easily available. These characteristics have opened the way for analyzing different natural compounds (such as genistein, apigenin, luteolin, lycopene, resveratrol, curcumin, and epigallocatechin-3-gallate) through clinical assays. In general, the preclinical studies of the agents mentioned, as well as others, have yielded positive results; however, when they have been examined in clinical assays, the results have been variable; that is, on occasion, they have shown positive results in different percentages, but in other cases, the data or results are not conclusive.This suggests that more complete and robust clinical assays should be carried out, taking care with the majority of the affectations in the selected population [7,210,215,216,217].

## 4. Conclusions

The present study (Part 2) synthesizes the most accurate evidence of the antigenotoxic capacity of some plants and/or vegetables and the main resin of natural origin gathered by bees against different toxic compounds that cause damage to genetic material. In the same fashion, the investigations presented confirm the use of these plants and resins in popular medicine to control genetic damage, existing within chronic degenerative diseases. The resin and plants described could offer novel alternatives to the limited therapeutic options that exist for disease reduction, whose development involves alterations in the genetic material (such as cancer). Thus, these vegetables and their phytochemicals should be considered in future studies.

In general, both articles (Part 1 and 2) identified and provided evidence of some phytochemicals with antigenotoxic activity, whose main mechanism of action is related to its antioxidant potential. Such a characteristic should motivate and promote the search for effective protective agents, which also must be further evaluated in pre-clinical and clinical assays to determine their safety and their chemopreventive capacity.

## Figures and Tables

**Table 1:** Main mechanisms of antimutagenic action.

Types of Mechanisms	Examples of Dietary Antimutagens
**EXTRACELLULAR**	
1. Inhibition of mutagen uptake	Dietary fibres, probiotics, grapefruit (naringenin).
2. Inhibition of endogenous formation *a) Inhibition of nitrosation* *b) Modification of the intestinal flora*	Vitamins (ascorbic acid), sulphur compounds (cysteine, glutathione). Prebiotics, probiotics.
3. Complexation and/or deactivation	Dietary fibres, chlorophyllin.
4. Favouring absorption of protective agents	Vitamin D3.
**INTRACELLULAR**	
5. Blocking or competition *a) Scavenging of reactive oxygen species* *b) Protection of DNA nucleophilic sites*	Mango (polyphenols), guava (gallocatechin) vitamins (β-carotene,α-tocopherol, ascorbic acid), pineapple, blueberries (anthocyanins). Ellagic acid, retinoids, polyamines.
6. Stimulation of trapping and detoxification in non-target cells	*N*-Acetyl cysteine.
7. Modification of transmembrane transport	Short chain fatty acids (caproate), dietary calcium.
8. Modulation of xenobiotic metabolising enzymes *a) Inhibition of promutagen activation* *b) Induction of detoxification pathways* *c) Inhibition of metabolic enzymes*	Isothiocyanates, monocyclic monoterpenoids (limonene, methol), flavonoids, wheat bran. Polyphenols, indoles, diterpene esters. Grapefruit (naringin, naringenin).
9. Modulation of DNA metabolism and repair	Cinnamaldehyde, vanillin.
10. Regulation of signaling pathways	Pomegranate (polyphenols), β-glucans.
11. Enhancement of apoptosis	Retinoids, flavonoids.
12. Maintenance of genomic stability	Vitamins (folic acid, B12), minerals (selenium, zinc), polyphenols.

Table modified from Ferguson et al. [10].

**Table 2:** Tests used in identifying genotoxic and antigenotoxic agents.

Prokaryote and Eukaryote Models Germinal Cell		
*In Vitro In Vivo*		
**I. GENE MUTATIONS**		
Bacteria (Ames assay, SOS chromotest) Yeast/Fungus (*S. cerevisiae assay*, *A. nidulans assay*)	Mouse spot test Somatic mutations and recombination test (SMART) DNA microarrays Serial analysis of gene expression (SAGE) Specific genes targeting	Recessive lethal Specific locus test Abnormalities of the sperm
**II. CHROMOSOME CHANGES**		
Fibroblast culture Lymphocyte culture Mouse lymphoma assay	Micronucleus assay (MN)	Dominant lethal Heritable translocations Cytogenetic sperm Aneuploidy
**III. INDICATORS BIOLOGICAL DAMAGE**		
Gene recombination Unscheduled DNA synthesis (UDS) assay	Comet assay Sister chromatid exchange (SCE) MN	Comet assay MN, SCE UDS assay

**Table 3:** Studies testing for antigenotoxic effects of broccoli.

Year	Main Objective and Conclusion	Type of Study	Assay Employed	Ref
**Forms of broccoli evaluated [fresh, juice, dialysate, extracts (water, ethanol, acetone), deep-frozen commercial broccoli, steamed before being consumed, and boiled or cooked with microwaves]**				
1980	Determination of the inhibitory potential of aqueous and acetone extractions from some common vegetables (including broccoli) against the mutagenicity induced by 3-methylcholanthrene and benzo[a]pyrene. The results showed that the extracts were antimutagenic in TA 100 strain and that the said capacity was proportional to the chlorophyllin content.	In vitro	AT	[52]
1988	Evaluation of the effect of aqueous dialysates of 16 vegetables and fruits on the mutagenicity of some carcinogens [3-amino-l-methyl-5H-pyrido (4,3-b) nature (Trp-P-2), benzo[a]pyrene, (BaP), aflatoxin B_l_ (AFB_1_), acrylamide, and N-methyl-N’-nitroso-N-nitrosoguanidine] in *Salmonella typhimurium* strain TA 100. All dialysates (including broccoli) inhibited the mutagenicity of Trp-P-2 and this antimutagenicity was maintained even after heating the dialysates at 100 °C for 20 min. The results also indicated that the apple dialysate was better at inhibiting the mutagenicity of BaP, AFB_1_, acrylamide, and N-methyl-N’-nitroso-N-nitrosoguanidine.	In vitro	AT	[53]
1994 1995	In summary, the researchers evaluated the antimutagenic potential of various vegetables [both in fresh form, as juices and extracts (n-hexane, dichloromethane, acetone, and 2-propanol)] against the toxic effect of 2-amino-3-methyl [4,5-f] -quinoline (IQ), 2-amino-3,4-dimethylimidazo [4,5-f] quinoline (MeIQ), and 2-amino-3,8-dimethylimidazo [4,5-f] quinoxaline (MeIQx) in strains, TA98 and TA100, of *Salmonella typhimurium*. They observed that both fresh vegetables and juices showed different levels, from weak to strong, of antimutagenic activity, unlike extracts where there was a greater antimutagenic effect, especially with n-hexane (96%), dichloromethane (64%), and acetone (44%). In both studies, they concluded that the peroxidase enzyme and the chlorophyll pigment present in broccoli and in other green vegetables, strongly contribute to this protective activity.	In vitro	AT	[54] [55]
1998 1999	Evaluation of the inhibitory effect of nine aqueous and ethanolic extracts from fruits and vegetables against the mutagenicity of N-nitrosodimethylamine (NDMA), N-nitrosopyrrolidine (NPYR), N-nitrosodibutylamine (NDBA), and N-nitrosopiperidine (NPIP). Both types of extracts showed an antimutagenic effect in the range of 50 microg/plate–2000 microg/plate, observing that broccoli was classified between the second and third places of effectiveness against all mutants.	In vitro	AT	[56] [57]
2007	The antimutagenic effect of broccoli flower head against mitomycin C (MMC) by the Ames Salmonella reverse mutation assay. Three strains (TA 98, TA102, and TA 1535) were challenged with the broccoli flower head ethanol extract (BFHEE) at concentrations of 23 mg/plate and 46 mg/plate. The conclusion of the experiment was that the BFHEE was not cytotoxic; on the contrary, the highest concentration showed a significant antimutagenic potential against MMC.	In vitro	AT	[58]
2008	This study is a continuation of the experiment conducted by Murugan SS et al. (2007). The investigation was on the effect of the BFHEE on the sister chromatid exchange (SCE) induced by MMC on cultured human peripheral blood lymphocytes. The enumeration of SCE in second division mitotic cells indicated that the BFHEE significantly reduced the SCE induced by MMC in both concentrations tested (200 microg/mL and 400 microg/mL).	In vitro	SCE	[59]
2002	Protective effect of broccoli (raw and cooked) against genotoxicity of 2-acetylaminofluorene (AAF) and 2-amino-1-methyl-6-phenylimidazo [4, 5-b] pyridine (PhIP) in V79 cells. The genotoxic activity of AAF and PhIP was strongly reduced in a dose-related manner by broccoli, demonstrating that protection of vegetables against genotoxicity of heterocyclic aromatic amines may take place within metabolically competent mammalian cells.	In vitro	CA	[60]
2006	The objective of the study was to determine whether selenized broccoli extracts decrease the hydrogen peroxide-induced DNA single-strand breaks measured in mouse hepatoma cells (Hepa 1c1c7 cells). The results showed that previous fertilization of broccoli with selenium was significantly effective (94%) to reduce the single-strand breaks to the genetic material. The data suggest that selenium is an important component for decreasing oxidative stress, but maximizing its content in the cultivation of broccoli might also stimulate its capacity to induce phase II detoxification proteins.	In vitro	CA	[61]
2006	This study examined the antigenotoxic and antioxidant properties of chlorophyll-rich methanol extracts of *Brassica oleracea*. The extract was highly effective in assays that measured ferric reducing/antioxidant power, oxygen radical absorbance property, and Fe^2+^/H_2_O_2_-DNA mediated nicking. Subsequently, by means of the “comet’ assay”, the methanol extract protected against H_2_O_2_-induced genotoxic damage in human HCT116 colon cancer cells. These findings provide support for the antigenotoxic and antioxidant properties of chlorophyll-rich extracts of *B. oleracea* through mechanisms that include inhibition of carcinogen activation and scavenging of reactive oxygen species.	In vitro	CA	[62]
2006	The objective was to investigate the differential effects of various selenium (Se) compounds and Se-enriched broccoli extracts on cell proliferation and the possible mechanism responsible for the Se-induced growth inhibition. C6 rat glial cells were incubated with graded concentrations up to 1000 nM of selenite, selenate, selenomethionine (SeM), Se-methyl-selenocysteine (SeMCys), high-Se broccoli (H-SeB) extract, or low-Se broccoli (L-SeB) extract for 24 h and 48 h. The comet assay indicated that there was no significant DNA single-strand break found for all Se treatments in C6 cells. In addition, the Se-induced proliferation inhibition may involve an H_2_O_2_-dependent mechanism with elevated cellular glutathione peroxidase (cGPX) activity. Both H-SeB and L-SeB inhibited C6 cell proliferation, but H-SeB was less inhibitory than L-SeB.	In vitro	CA	[63]
2010	This study was a continuation of the experiment conducted by Edenharder R. et al. (2002). Chinese hamster lung fibroblasts, genetically engineered for the expression of rat cytochrome P450 dependent monooxygenase 1A2 and rat sulfotransferase 1C1 (V79-rCYP1A2-rSULT1C1 cells), were utilized to detect protective effects of plant-based beverages against the genotoxicity induced by 2-acetylaminofluorene (AAF) or 2-amino-1-methyl-6-phenylimidazo [4,5-b]pyridine (PhIP). The genotoxic activity of PhIP was strongly reduced in a dose-related manner by broccoli (raw and cooked). Similarly, the genotoxicity of AAF showed a reduction, although in a lower potency by cooked broccoli. These results are suggestive for enzyme inhibition (possibly involves CYP1A2) as a protection mechanism induced by the complex chemical mixtures present in the plant-based beverages.	In vitro	CA	[64]
2012	*B. oleracea* L. var. costata leaves and *Pieris brassicae* L. larvae aqueous extracts were assayed for their potential characteristics to prevent/induce DNA damage. Using the comet assay, none of the extracts revealed to be genotoxic by itself, and both showed protection, more advisable on larvae extracts, against MMS-induced genotoxicity. As for the genotoxic/antigenotoxic effects of Brassica vegetables, they are commonly attributed to isothiocyanates. The extracts were screened in search of these compounds by means of a headspace-solid-phase microextraction/gas chromatography-mass spectrometry. These findings demonstrate that both extracts could be useful against damage caused by genotoxic compounds; with the larvae extract being the most promising.	In vitro	CA	[65]
2012	Because cruciferous species are usually processed before eating and the real impact of cooking practices on their bioactive properties is not fully understood, Ferrarini L. et al., evaluated the effect of common cooking practices (boiling, microwaving, and steaming) on the biological activities of broccoli, cauliflower, and Brussels sprouts. The objective was to determine and compare the chemoprotective capacity of fresh and cooked vegetable extracts against oxidative DNA damage induced by 3-(4,5-dimethylthiazol-2-yl)-5-(3-carboxymethoxyphenyl)-2-(4-sulfophenyl)-2H-tetrazolium in HT-29 human colon carcinoma cells. The results indicated that both types of vegetables showed a protective activity comparable to vitamin C. In conclusion, the cooking methods applied did not alter the antioxidant effect of raw vegetables.	In vitro	CA	[66]
2006	The purpose of this study was to examine the antimutagenic and antigenotoxic effect of vegetable homogenates (broccoli, cauliflower, red cabbage, onion, garlic) on the damage produced by AFB_1_, 2-amino-3-methylimidazo [4,5, -f] quinolin (IQ), and N-nitroso-N-methylurea (MNU). The Ames test, in vivo micronuclei, and the comet assay showed that all homogenates contained clear antigenotoxic activities. Only, in the Ames test, the effect of some phytochemicals against the direct mutagen, MNU, was lower in comparison to the indirect mutagens, AFB_1_ and IQ.	In vivo	CA & MN	[67]
2006	The analysis was on the chemoprotective effect of broccoli juice treated under high pressure (500 MPa during 10 min) against the damage produced by N-nitroso-N-methylurea (MNU) in BALB/C mice. After administering broccoli juice (0.2 mL/10 g) for 14 days and a unique application of the mutagen, a statistically significant decrease in the number of micronuclei induced by the MNU was obtained. As a conclusion, substances that favor chemoprotective capacity, such as vitamins, polyphenolic componds, glucosinates, etc., can be preserved in broccoli juice treated at high pressure.	In vivo	MN	[68]
2008	These researchers evaluated the protective effect of mustard leaf (*Brassica campestris*), a popular Indian cruciferous vegetable, against chromosomal damage and oxidative stress induced by gamma-radiation, cyclophosphamide (CP), and urethane (URE) through the in vivo bone marrow micronucleus test. A pretreatment with 50 mg/kg-250 mg/kg of mustard leaf extract (MLE) for seven days significantly reduced the frequencies of micronuclei induced by gamma-radiation, CPH, and URE. The protective effect against chromosomal damage was associated with the modulation of lipid peroxidation and with an increase in GSH and the GSH-dependent enzyme, glutathione S-transferase (GST).	In vivo	MN	[69]
2009	The objective was to evaluate the protective effect of broccoli intake in smokers and non-smokers. 20 healthy young people (10 smokers and 10 nonsmokers) were randomized in a crossover design and received a 200 g portion of brocolli or maintained a controlled diet for 10 days each. Blood samples were collected at 0, 10, 30, and 40 days to evaluate the DNA damage. The ex vivo protection of DNA damage induced by H_2_O_2_ and damage of endogenous DNA were evaluated in lymphocytes by the comet assay. Strand breaks decreased significantly after the broccoli diet, both in smokers and nonsmokers.	Clinical study	CA	[70]

Ames test (AT), sister chromatid exchange (SCE), micronucleus (MN), comet assay (CA), methyl methanesulfonate (MMS).

**Table 4:** Studies testing of the antigenotoxic effects of broccoli main phytochemicals.

Year	Main Objective and Conclusion	Type of Study	Assay Employed	Ref
**Main phytochemicals [sulforaphane (SUL), phenethyl isothiocyanate (PEITC), allyl isothiocyanate (AITC), and indole-3-carbinol (I3C)]**				
1996	The study tested the sulforaphane (SUL) capacity to inhibit the genotoxicity of N-nitrosodimethylamine (NDMA) in the TA100 strain of Salmonella typhimurium. The results showed that SUL reduces the mutagenicity of NDMA at concentrations of 0.8 microM and suggest that its mechanism of action involves the inhibition of cytochrome P450 isoenzyme 2E1 (CYP2E1).	In vitro	AT	[41]
2003	The aim was to determine the antimutagenic potential of sulforaphane (SUL) against different heterocyclic amines present in cooked foods. The use of strains, TA98 and TA100, of Salmonella typhimurium demonstrated that SUL is a potent inhibitor. Approximately 60% of the mutagenicity was induced by 2-amino-3-methylimidazo [4,5-f] quinoline (IQ), 2-amino -3,4-dimethylimidazo [4,5-f] quinoline (MeIQ), and 2-amino-3,8-dimethylimidazo [4,5-f] quinoxaline (MeIQx).	In vitro	AT	[42]
2009	This study was a continuation of the experiment conducted by Shishu and Kaur (2003). In this case, the antimutagenic potential of SUL (extracted from broccoli) and sulforaphane (found in the radish) was compared and evaluated against the same heterocyclic amines present in cooked foods. The use of the same strains of S. typhimurium demonstrated that both isothiocyanates were potent inhibitors of the mutagenicity induced by all the mutagens tested. However, sulforaphane showed a greater chemoprotective activity than SUL did.	In vitro	AT	[43]
1998	The study demonstrated that in human liver cells, T5-2E1 and T5-1A2, the N-nitrosodimethylamine (NDMA) and 2-amino-3-methylimidazo [4,5-f] quinoline (IQ) generated DNA strand breaks. The comet assay showed that SUL markedly reduced the DNA damage induced by these mutagens; likewise, the results suggest that the inhibition of the said genotoxicity was mediated by the action of CYP2E1 and CYP1A2 isoenzymes, which contributes to a relevant chemopreventive activity.	In vitro	CA	[44]
2001	The study showed that a pretreatment with SUL in LS-174 cells before exposing them to BaP for 24 h reduced the number of single-strand DNA breaks (approximately 30%) generated by the carcinogen. This suggests that indoles and isothiocyanates (ITCs) from some cruciferous vegetables (including broccoli) can stimulate apoptosis in human colon adenocarcinoma and induce DNA protection.	In vitro	CA	[45]
2004	The aim was to evaluate the ability of indole-3-carbinol (I3C) to reduce DNA damage generated by N-methyl-N’-nitro-N-nitrosoguanidine (MNNG) in cultured Chinese hamster lung fibroblast cells (CH V-79). The result was that MNNG produced DNA single strand breaks in a dose- and time-dependent manner, as was determined by the increase in the tail moment of the comet. In contrast, when the cells were pretreated with I3C, a significant protection was induced in the DNA. Thus, concluding that I3C can be considered a relevant chemopreventive agent capable of inducing the DNA repair.	In vitro	CA	[46]
2006	The evaluation was on the protective effect of the three most common isothiocyanates (ITCs) of broccoli (phenethyl isothiocyanate (PEITC), allyl isothiocyanate (AITC), and indole-3-carbinol (I3C)) towards DNA damage induced by N-nitrosamines in HepG2 cells. The results showed that none of the concentrations used of the ITCs caused DNA damage per se. The three ITCs showed a genoprotective effect of oxidative damage induced by N-nitrosopyrrolidine (NPYR) and/or N-nitrosodimethylamine (NDMA); however, the most important protection corresponded to I3C and PEITC against NPYR.	In vitro	CA	[47]
2008	The study was on the protective effect of isothiocyanates (ITCs) alone or in combination with vitamin C towards oxidative DNA damage induced by N-nitrosodibutylamine (NDBA) and/or N-nitrosopiperidine (NPIP) in HepG2 cells. The conclusions were that both the PEITC and the I3C showed a weak protective effect (27%) against the NDBA and NPIP. In contrast, HepG2 cells treated with the combination of vitamin C and each of the ITCs presented a stronger genoprotection of oxidative damage induced by these carcinogens. This evidence suggests that a possible mechanism of action of ITCs (alone or in combination with vitamins) could be regulating the bioactivation of NDBA and NPIP through cytochrome P450.	In vitro	CA	[48]
2009	Due to the evidence that exposure to certain pesticides represents a potential risk to human health, the antigenotoxic capacity of SUL against endosulfan, chlorpyrifos, and thiram in human lymphocytes was assessed by the comet assay. The mixture of pesticides at an environmentally relevant concentration (5 microM each) produced DNA damage in the lymphocytes while with a pre-incubation and co-incubation of SUL, a significant genoprotection was observed in a concentration dependent manner(between 10 microg/mL–20 microg/mL). These data suggest that the exposure to low levels of these pesticide mixtures can induce DNA damage, and the presence of SUL in the diet can reduce the incidence of this genetic damage, especially in farm workers.	In vitro	CA	[49]
2013	Protecting the eye lens against oxidative stress is of great importance to delay the onset of cataracts. Sulforaphane may be a good strategy to provide cytoprotection against oxidative stress. Therefore, the ability of SUL to perform this function on the lens cells was evaluated and its potential to delay the onset of the cataract was established. The comet assay determined the level of DNA strand breaks in human lens epithelial cell line, FHL124. The exposure of 30 μM of H_2_O_2_ to these cells caused a reduction in cell viability and an increase in cytotoxicity; whereas a pre-treatment with SFN inhibited these effects and significantly reduced the DNA strand breaks induced by H_2_O_2_.	In vitro	CA	[50]
1997	Using the micronucleus assay, the mutagenicity of the pesticide, propoxur, and its inhibition was determined by the administration of indole-3-carbinol (I3C) in Swiss mice. Intraperitoneal administration of propoxur (25 mg/kg) induced MN formation in bone marrow cells after a 48h exposure. In contrast, the application of I3C (500 mg/kg body weight) significantly inhibited the genotoxicity.	In vivo	MN	[51]

Ames test (AT), micronucleus (MN), and comet assay (CA).

**Table 5:** Main components of the essential oil of chamomile [75].

Component	RT^2^	Area in the Plant (%)
(*E*)-β- farnesene	38.46	28.17
Germacrene-*D*	39.23	2.19
Unidentified sesquiterpene	40.07	1.40
Unidentified sesquiterpene	41.17	0.78
(*Z,E*)-α- farnesene	41.35	1.59
Unidentified sesquiterpene	48.52	0.71
α-bisabolol oxide A	54.46	41.77
α-bisabolol oxide B	49.28	4.31
α-bisabolol oxide	50.65	5.30
α-bisabolol	51.18	2.31
Chamazulene	52.80	2.39
1,6-dioxaspiro [4,4] non-3-ene,2-(2,4 hexadyn-1-ylidene)	60.73	2.19
Hexatriacontane	67.49	0.50

RT^2^: Retention time obtained with gas chromatography.

**Table 6:** Studies carried out with the comet assay that evaluates the antigenotoxic effect of cocoa catechins.

Year	Main objective and Conclusion	Type of Study	Type of Procyanidin	Ref
2008 2009	The aim was to determine the protective effect of CAT and EPI against N-nitrosamines and benzo[a]pyrene-induced DNA damage (strand breaks and oxidized purines/pyrimidines) in HepG2 human hepatoma cells. Both polyphenols in concentrations of 10 microM decreased the DNA strand breaks (approximately 30%) generated by the action of mutagens.	In vitro	CAT & EPI	[100] [101]
2011	This study analized the effect of procyanidins on the oxidative DNA damage induced by some heterocyclic amines in human hepatoma cells (HepG2). The three amines (8-MeIQx, 4,8-diMeIQx, PhIP) increased the purines and pyrimidines oxidized; and, consequently, the number of breaks of DNA strands. In contrast, the action of procyanidins reduced the effect.	In vitro	CAT & EPI	[102]
2013	This in vivo study evaluated the influence of EPI on the genotoxicity induced by etoposide in bone marrow cells of male rats. The results showed that EPI significantly reduced the DNA strand break produced by this drug.	In vivo	EPI	[103]
2017	The objective was to isolate and evaluate the antigenotoxic capacity of the main phytochemicals of *Paliurus spina-christi Mill* in a Chinese hamster lung fibroblasts (V79) cell line. The result was that the methanol extract of this typical Turkish fruit did not induce DNA damage. In contrast, there was a significant reduction of isolated procyanidins on DNA damage induced by H_2_O_2_.	In vitro	CAT & GALL	[104]

Epicatechin (EPI), catechin (CAT), gallocatechin (GALL).

**Table 7:** Latest research on the antigenotoxic capacity of the main phytochemicals of the essential oil of *Laurus nobilis* L.

Year	Main objective and Conclusion	Assay Employed	Type of Study	Ref
Eucalyptol (EUC)				
2007	This study examined the possible protective effect of EUC against the DNA damage induced by H_2_O_2_ in human leukemic K562 cells. The results were not fully conclusive. No significant decrease in the level of breaks of single strands of DNA was observed.	CA	In vitro	[125]
2011	The aim was to examine the protective potential of plant monoterpenes against 4-Nitroquinoline 1-oxide (4NQO)-induced genotoxicity in the Vero cell line. Incubation of 4NQO-pretreated Vero cells with EUC resulted in a significant reduction of the tail moment. However, higher concentrations of monoterpenes induced DNA strand breaks. As in the previous study, the results are not fully conclusive and suggest that EUC can stimulate error-free DNA repair processes and may act as bioantimutagen.	CA	In vitro	[126]
Eugenol (EUG)				
2010	This study investigated the preventive effect of EUG on thioacetamide (TA) -induced liver injury. The pretreatment of EUG decreased the elevated expression of the COX-2 gene and DNA strand breaks induced by TA. These findings suggest that EUG reduces the genotoxic effects of TA in the liver.	CA	In vivo	[127]
2014	The evaluation was on the modulating effect of EUG (0.31 μg/mL, 0.62 μg/mL, 1.24 μg/mL, and 2.48 μg/mL) on DNA damage induced by doxorubicin (DXR) in mouse peritoneal macrophages. The data were confusing because EUG showed both genotoxic and antigenotoxic potential. These results suggest that EUG can modulate the DNA damage induced by DXR, but it should be cautiously used and investigations should be extended to confirm whether they could induce a primary DNA damage.	CA	In vitro	[128]
2004	Three doses of EUG (75 mg/kg,150 mg/kg, and 300 mg/kg) were administered to Swiss albino mice before being exposed to 1.5 Gy of gamma radiation. The results showed a significant reduction in the frequency of micronucleated polychromatic erythrocytes (MNPE) with the three doses used; in addition, this decrease was sustained up to 72 h after the irradiation. These data revealed that the EUG exerted a significant protection against oxidative stress and suggest a radioprotective potential.	MN	In vivo	[129]
2001	The aim was to determine the antigenotoxic potential of EUG against the damage produced by cyclophosphamide (CP), procarbazine (PCB), N-methyl-N’-nitro-N-nitrosoguanidine (MNNG), and urethane (URE). The oral administration of EUG (50 mg/kg–500 mg/kg) before injecting the genotoxins showed a dose-dependent protective effect. It also confirmed that the EUG is not a micronucleus-inducing agent in mouse bone marrow.	MN	In vivo	[130]
Linalool (LIN)				
2009	The results of this study suggest that LIN can be considered an important antioxidant agent, because it is a natural monoterpene capable of reducing DNA damage induced by t-butyl hydroperoxide in two types of cell lines (human hepatoma (HepG2) and human B lymphoid cells (NC-NC)) by approximately 50%.	CA	In vitro	[131]
2017	This study analyzed the preventive effect of LIN against the oxidative imbalance induced by ultraviolet-B radiation (UVB) in human skin cells (HDFa). LIN significantly reduced the formation of 8-deoxy guanosine mediated by UVB. This result suggests that this natural monoterpene can be considered a photoprotective agent for preventing the formation of reactive oxygen species (ROS).	CA	In vitro	[132]
Geraniol (GER)				
2007	There is no concrete evidence on the antigenotoxic capacity of GER. The results of this study suggest that it is not a clastogenic agent; because it does not increase the frequency of MN in mouse bone marrow.	MN	In vitro	[133]

Micronucleus (MN) and comet assay (CA).

**Table 8:** Major compounds identified in propolis resin [18].

Chemical Groups	Compounds
Alcohols	Benzyl alcohol, cinnamyl alcohol, glycerol, α-glycerol phosphate, hydroquinone, isobutenol, phenethyl alcohol
Aldehydes	Benzaldehyde, caproic aldehyde, p-hydroxybenzaldehyde, isovanillin, vanillin
Aliphatic acids and aliphatic esters	Acetic acid, angelic acid, butyric acid, crotonic acid, fumaric acid, isobutyric acid, methylbutyric acid, isobutyl acetate, isopentyl acetate
Amino acids	Alanine, β-alanine, α-aminobutyric acid, γ-aminobutyric acid, arginine, asparagine, aspartic acid, cysteine, glutamic acid, glycine, histidine, isoleucine, leucine, lysine, methionine, ornithine, phenylalanine, proline, serine, threonine, tryptophan, tyrosine, valine
Aromatic acids	Benzoic acid, caffeic acid, cinnamic, coumaric, acid, ferulic acid, gallic acid, gentisic acid, hydroxycinnamic acid, p-hydroxybenzoic acid, isoferulic acid, 4-methoxy cinnamic acid, salicylic acid, vanillic acid
Aromatic esters	Benzyl acetate, benzyl benzoate, benzyl caffeate, benzyl coumarate, benzyl ferulate, benzyl isoferulate, benzyl salicylate, butenyl caffeate, butyl caffeate, cinnamyl benzoate, cinnamyl caffeate, ethyl benzoate
Chalcones	Alpinetin chalcone, naringenin chalcone, pinobanksin chalcone, pinocembrin chalcone, sakuranetin chalcone
Flavanones	Naringenin, pinobanksin, pinobanksin-3-acetate, pinobanksin-3-butyrate, pinobanksin-3-hexanoate, pinobanksin-3-methyl ether, pinobanksin-3-pentanoate
Flavones and flavonols	Acacetin, apigenin, apigenin-7-methyl ether, galagin, galagin-3-methyl ether, izalpinin, isorhamnetin, kaempferol, quercetin, ramnetin, ramnocitrin
Waxy acids	Arachid acid, behenic acid, cerotic acid, lauric acid, linoleic acid, lignoceric acid, montanic acid, myristic acid, oleic acid, palmitic acid, stearic acid
Ketones	Acetophenone, dihydroxy-acetophenone, methylacetophenone
Terpenoids and other compounds	α-acetoxybetulenol, β-bisabolol, 1,8-cineole, α-copaene, cymene, limonene, styrene, xanthorreol, naphthalene, sesquiterpene alcohol, sesquiterpene diol
Steroids	Calinasterol acetate, b-dihydrofucosterol acetate, ucosterol acetate, stigmasterol acetate
Sugars	Fructofuranose, α-D-glucopyranose, β-D-glucopyranose

**Table 9:** Studies testing for antigenotoxic effects of propolis.

Year	Authors	Main Objective, Results, and Conclusion	Ref
**Comet assay in vivo**			
2005	de Lima et al.	Evaluation of the modifying effect of an aqueous extract of propolis (AEP) on the formation of aberrant crypt foci (ACF) and DNA damage induced by 1,2-dimethylhydrazine (DMH) in the colon of Wistar rats. The AEP was orally administered at approximate doses of 12 mg/kg, 34 mg/kg, 108 mg/kg, and 336 mg/kg of body weight/day. Subsequently, the animals received different injections of DMH. The results showed that the simultaneous administration of both compounds reduced the induction of DNA damage in the colon. However, AEP had no effect on the formation of ACF.	[188]
2008	Benkovic et al.	The protective effect of a water-soluble derivate of propolis (WSDP) and some flavonoids (including caffeic acid, chrysin, and naringin) against damage caused by two doses of gamma irradiation was analyzed in this study. Like the previous study, the data indicated that all compounds administered before irradiation protect animals from lethal effects of whole-body irradiation and diminish primary DNA damage in their white blood cells. The WSDP showed the best radioprotective effect.	[192]
2008	Benkovic et al.	The objective was to analyze the radioprotective effects of the ethanolic extract of propolis (EEP) and quercetin on the white blood cells of mice irradiated with gamma rays. Irradiation was performed using a gamma-ray source [(60) Co], and the absorbed dose was 9 Gy. The efficacy of the compounds was evaluated intraperitoneally at a dose of 100 mg/kg for three consecutive days before and/or after irradiation. The results showed that propolis and quercetin protected these cells from the lethal effect of irradiation and decreased primary DNA damage.	[193]
2009	Benkovic et al.	The aim of this study was to assess radioprotective effects of quercetin and the ethanolic extract of propolis (EEP) in CBA mice exposed to a single radiation dose (4 Gy) of gamma radiation. Similar to the two previous studies, the mice were treated daily with 100 mg/kg of quercetin or EEP for three consecutive days before or after gamma irradiation. The leukocyte count was determined in blood drawn from the tail vein, and DNA in these cells was assessed using the comet assay. Animals pretreated with the compounds were less sensitive to irradiation. Those that received the therapy after irradiation showed a slight, but not significant, increase in the total leukocyte count compared to the negative control. EEP and quercetin were confirmed not to be genotoxic for non-irradiated mice.	[194]
**Comet assay in vitro**			
2011	Aliyazicioglu et al.	The analysis was on the antigenotoxic potential of propolis extracts in foreskin fibroblast cells against oxidative damage induced by hydrogen peroxide. The results showed a significant decrease in DNA damage induced by H_2_O_2_ in the cultures pretreated with the extract. These data suggest that the antigenotoxicity of propolis may occur under different mechanisms, including the antioxidant activity of the phenolic components present in the extract.	[189]
2016	Yalcinet al.	The objective was to evaluate the protective effect of ethanolic extract of propolis (EEP) against γ-ray-induced DNA damage on fibroblast cells. Initially, the cells were pretreated for 15 min and 30 min with three different concentrations of EEP (100 μg/mL, 200 μg/mL, and 300 μg/mL); subsequently, they were exposed to 3 Gyγ-rays. The results showed a significant decrease in γ-ray-induced DNA damage on cells treated with EEP. It was concluded that EEP might have radioprotective activity.	[190]
**Micronucleus, chromosome aberrations, and** **comet assay** **in vitro**			
2008	Benkovic et al.	Using three different cytogenetic tests, the protective effect of propolis and quercetin against damage to the genetic material induced by gamma radiation in a culture of human leukocytes was evaluated. The results suggested that both compounds have a low toxicity profile in in vitro conditions. Likewise, it was observed that they could be considered radioprotective agents, because they reduced the levels of DNA damage in leukocytes irradiated with gamma rays in the three types of tests evaluated. It was confirmed that propoleo was the most effective and that it can have different mechanisms of protective action.	[191]
2009	Benkovic et al.	This in vitro study aimed to evaluate the possible radioprotective effects of water-soluble derivate of propolis (WSDP), caffeic acid, chryrin, and naringin on gamma-irradiated human white blood cells. Using the same three tests as in the previous study, it was confirmed that none of the tested compounds induced significant genotoxicity; on the contrary, they offered a measurable protection against DNA damage. It was observed that the WSDP was the most effective in reducing levels of cytogenetic DNA damage in white blood cells.	[195]
**Micronucleus and comet assay in vitro**			
2016	Roberto et al.	In this work, the genotoxic and antigenotoxic potential of the ethanolic extracts of Brazilian green propoleo and B. dracunculifolia in mammalian cells (HTC cells) was evaluated. After evaluating the exposure of the cells to each extract, individually and in combination with the MMS, the results showed that no extract was genotoxic; on the contrary, they exerted a significant reduction in DNA damage. The experiment carried out with a pre-incubation period was more effective than without the incubation test, showing that the tested extracts were able to inactivate the mutagen before it could react with the DNA.	[206]
**Micronucleus and comet assay in vivo**			
2013	Oršolić et al.	The study was on the antitumor, chemopreventive, and immunostimulative effects of local chemoimmunotherapy and hyperthermal intraperitoneal chemotherapy (HIPEC) in a mouse-bearing Ehrlich ascites tumor (EAT). In this case, mice were treated with WSDP at a dose of 50 mg/kg, seven and three days before implantation of EAT cells, whereas the cisplatin (CIS) was injected three days after implantation of EAT cells. The combination of WSDP plus CIS resulted in tumor growth inhibition and increased the survival of mice at approximately 115%. The study also confirmed that WSDP reduced the cisplatin toxic and genotoxic effect to normal cells without affecting cisplatin cytotoxicity on EAT cells.	[196]
**Micronucleus assay in vivo**			
2010	Türkez et al.	With the purpose of determining the effectiveness of propolis in the modulation of the genotoxicity induced by aluminum chloride (AlCl (3)) and the hepatotoxicity in the liver of rats, in a group of animals, a dose of the natural resin (50 mg/kg) plus AlCl (3) was administered orally daily for 30 days. At the end of the experiment, the histological alterations in the liver were investigated and their hepatocytes (HEP) were isolated to count the number of micronucleated hepatocytes (MNHEPs). This simultaneous treatment significantly modulated the pathological damages generated by aluminum chloride (sinusoidal dilatation, congestion of the central vein, lipid accumulation, and infiltration of lymphocytes) and reduced the frequency of MNHEPs. In conclusion, propolis may have an anticlastogenic effect and might antagonize the toxicity of AlCl (3).	[199]
2010	Oršolić et al.	Considering previous studies, where WSDP, quercetin, and naringin showed their effectiveness to reduce the DNA damage induced by gamma irradiation in white blood cells, the same group of researchers evaluated this property against the toxicity induced by irinotecan (antineoplastic derived from camptothecin and extracted from the Chinese tree, *Camptotheca acuminata*). On this occasion, they determined that propolis and some flavonoids could reduce the cyto and genotoxicity induced by irinotecan in cells of mice with Ehrlich ascites tumors (EAT); and at the same time, protect normal blood, liver, and kidney cells. The pre-treatment with propolis and/or flavonoides resulted in a substantial inhibition of the growth of EAT cells and significantly reduced the frequency of micronuclei. These results suggest that propolis have a significant immunomodulatory effect and can decrease the toxicity of the antineoplastic in normal cells.	[198]
2014	Dornelas et al.	The objective of this study was to evaluate the anticlastogenic effect of green propolis and L-lysine on the damage produced by the carcinogen, BBN [n-butyl-n (4-hydroxybutyl) nitrosamine], in erythroblasts (bone marrow) and leukocytes (peripheral blood). After 40 weeks of treatment with the evaluation compounds, the animals were anesthetized and subjected to femoral bone marrow aspiration and blood collection from the aorta to quantify the frequency of MN. The study confirmed that both propolis (150 mg/kg) and L-lysine (300 mg/kg), alone or in combination, were not genotoxic by themselves toward the cells evaluated; on the contrary, the dosage and timing were effective in protecting against the genotoxicity of BBN.	[207]
2014	Oršolić et al.	The analysis was on the inhibitory effect of an ethanolic extract of propolis (EEP) on the skin irritation, oxidative stress, and inflammatory response induced by n-Hexyl salycilate (HXS) or Di-n-propyl disulfide (PPD) in mice. The inflammation process was monitored by histopatological assessment of the skin, total number of inflammatory cells in the peritoneal cavity, macrophage-spreading index, and frequencies of micronucleated reticulocytes, lipid peroxidation, and glutathione assay in the skin. The topical application of EEP reduced the lipid peroxidation, the total number of inflammatory cells in the peritoneal cavity, and functional activity of macrophages. A significant decrease in the number of micronucleated reticulocytes was observed. These results demonstrate that a topical application of EEP may improve psoriatic-like skin lesions by suppressing the functional activity of macrophages and ROS production.	[197]
**Micronucelus assay in vitro**			
2012	Türkez et al.	With the goal to determine the effectiveness of this natural resin in alleviating the 2,3,7,8-tetrachlorodibenzo-p-dioxin (TCDD)-induced toxicity in rat hepatocytes, three concentrations of propolis (25 μM, 50 μM, and 100 μM) were added in combination with TCDD to a primary culture of hepatocytes. The cell viability, total antioxidant capacity (TAC), levels of oxidative stress (TOS), and DNA damage were further quantified. The evidence indicated that in cultures treated with TCDD only, the cell viability and the level of TAC decreased, while the frequency of micronucleated hepatocytes (MNHEPs) increased. A contrary situation was present in cultures with a combination of TCDD and propolis, in which the resin modulated its toxic effects and significantly reduced the number of MNHEPs.	[200]
2014	Santos et al.	Brazilian researchers evaluated the effect of propolis (type AF-08) on the genotoxicity, cytotoxicity, and clonogenic death of Chinese hamster ovary (CHO-K1) cells irradiated with (60) Co gamma-radiation. The MN test revealed that AF-08 alone (5 μg/mL–100μg/mL) was not genotoxic and that a low concentration of it (30μg/mL) reduced the damage induced by radiation in the DNA. On the other hand, analysis of cytotoxicity showed that a concentration of 50μg/mL presented a significant proliferative effect when associated with radiation, decreasing the percentage of necrotic cells. Concerning the clonogenic capacity, AF-08 also evidenced a significant stimulating effect on cell proliferation. Together, these data suggest that propolis AF-08 might be potentially radioprotective.	[208]

## Note

Long and successful life to the Fourth Mexican transformation (E. Madrigal-Bujaidar and I. Álvarez-González).
